# Therapeutic ACPA inhibits NET formation: a potential therapy for neutrophil-mediated inflammatory diseases

**DOI:** 10.1038/s41423-020-0381-3

**Published:** 2020-03-20

**Authors:** Renato G. S. Chirivi, Jos W. G. van Rosmalen, Maarten van der Linden, Maximilien Euler, Gonny Schmets, Galina Bogatkevich, Konstantinos Kambas, Jonas Hahn, Quinte Braster, Oliver Soehnlein, Markus H. Hoffmann, Helmuth H. G. van Es, Jos M. H. Raats

**Affiliations:** 1 ModiQuest B.V., Oss, The Netherlands; 2 Citryll B.V., Oss, The Netherlands; 3 Department of Internal Medicine 3 – Rheumatology and Immunology, University Hospital Erlangen, Friedrich-Alexander-Universität Erlangen-Nürnberg and University Hospital Erlangen, Erlangen, Germany; 4 Department of Medicine, Division of Rheumatology and Immunology, Medical University of South Carolina, Charleston, SC USA; 5 Laboratory of Molecular Hematology, Democritus University of Thrace, Alexandroupoli, Greece; 6 Institute for Cardiovascular Prevention (IPEK), Ludwig-Maximilians-University Munich, Munich, Germany; 7 German Center for Cardiovascular Research (DZHK), Partner Site Munich Heart Alliance, Munich, Germany; 8 Department of Physiology and Pharmacology, Karolinska Institutet, Stockholm, Sweden

**Keywords:** Neutrophil Extracellular Traps, Autoimmunity, Citrullination, Therapeutic Antibody, NET inhibition, Translational immunology, Autoimmunity

## Abstract

Excessive release of neutrophil extracellular traps (NETs) is associated with disease severity and contributes to tissue injury, followed by severe organ damage. Pharmacological or genetic inhibition of NET release reduces pathology in multiple inflammatory disease models, indicating that NETs are potential therapeutic targets. Here, we demonstrate using a preclinical basket approach that our therapeutic anti-citrullinated protein antibody (tACPA) has broad therapeutic potential. Treatment with tACPA prevents disease symptoms in various mouse models with plausible NET-mediated pathology, including inflammatory arthritis (IA), pulmonary fibrosis, inflammatory bowel disease and sepsis. We show that citrulline residues in the N-termini of histones 2A and 4 are specific targets for therapeutic intervention, whereas antibodies against other N-terminal post-translational histone modifications have no therapeutic effects. Because citrullinated histones are generated during NET release, we investigated the ability of tACPA to inhibit NET formation. tACPA suppressed NET release from human neutrophils triggered with physiologically relevant human disease-related stimuli. Moreover, tACPA diminished NET release and potentially initiated NET uptake by macrophages in vivo, which was associated with reduced tissue damage in the joints of a chronic arthritis mouse model of IA. To our knowledge, we are the first to describe an antibody with NET-inhibiting properties and thereby propose tACPA as a drug candidate for NET-mediated inflammatory diseases, as it eliminates the noxious triggers that lead to continued inflammation and tissue damage in a multidimensional manner.

## Introduction

Neutrophils are the most abundant type of leukocytes in human blood, contribute to the first line of defense and use their extensive armory to protect the host against infection. Neutrophils kill microbes via phagocytosis, generation of reactive oxygen species (ROS), or release of their granular contents. A more recently described antimicrobial function of neutrophils is neutrophil extracellular trap (NET) formation.^1^ NETs confine and efficiently eliminate pathogens and have been shown to protect mice and humans against bacterial^2,3^ and fungal^4^ infections. Despite their importance in host defense, aberrant and prolonged NET release is associated with the pathophysiology of many acute and chronic inflammatory disorders (reviewed in refs. ^5–9^). In particular, incomplete clearance of NETs contributes to vascular injury, which leads to tissue damage and organ failure or even death.^10^ NETs have been shown to block tissue repair signals, leading to impaired wound healing in diabetes,^11^ while activation of the clotting system by NETs occludes blood vessels in thrombosis.^12^ In addition, antimicrobial proteins and histones that are present in NETs are highly cytotoxic and induce endothelial dysfunction in systemic lupus erythematosus (SLE),^13,14^ vasculitis,^15^ and sepsis.^16^ Furthermore, NETs are a source of autoantigens and trigger autoimmunity, which is associated with the production of autoantibodies against various NET components in rheumatoid arthritis (RA),^17^ small-vessel vasculitis (SVV),^18^ antiphospholipid syndrome (APS),^19^ and SLE.^19,20^

The formation of ROS via nicotinamide adenine dinucleotide phosphate (NADPH) oxidase complex 2, myeloperoxidase (MPO), or mitochondria, together with the translocation of neutrophil elastase (NE) and MPO to the nucleus, is a key mechanism of NET release. Moreover, conversion of arginine to citrulline on histones by peptidyl arginine deiminase 4 (PAD4) is necessary to promote chromatin decondensation and the subsequent release of NETs in the extracellular environment.^21^ Interestingly, pharmacological or genetic inhibition of PAD4 disrupts NET release and reduces pathology in various murine disease models, including atherosclerosis,^22^ inflammatory arthritis (IA),^23^ and SLE.^24,25^ Therefore, NETs are potential therapeutic targets for different acute and chronic inflammatory disorders.

We previously engineered a therapeutic anti-citrullinated protein antibody (tACPA) that specifically binds to citrulline at position 3 (Cit3) in histone 2A (citH2A) and 4 (citH4).^26^ This antibody showed strong anti-inflammatory activities in an acute collagen antibody-induced arthritis (CAIA) mouse model of IA.^26^ In the current study, we further investigated the therapeutic characteristics of tACPA and demonstrated that tACPA inhibits murine and human NET formation and binds to NETs in vitro and in vivo, potentially initiating clearance by macrophages. Using a preclinical basket approach, we showed the therapeutic and prophylactic potential of tACPA in diseases in which NETs are the drivers of, or contribute to, the pathology.

## Results

### Different tACPA molecules used in this study

Our previous work demonstrated that tACPA derived from RA patients (h-tACPA), as well as hybridoma-derived tACPA (m-tACPA), exhibits strong anti-inflammatory activity in a CAIA mouse model of IA and specifically targets Cit3 in the N-termini of citH2A and citH4,^26^ with no cross-reactivity with N-terminal homo-citrullinated and acetylated H2A and H4 (Supplementary Fig. 1). Because m-tACPA had better characteristics and efficacy compared with those of h-tACPA, we continued with m-tACPA and engineered the molecule step-by-step, leading to improved intermediate chimeric/human tACPA molecules that have distinct features. Each time an improved molecule became available, we used it in our experiments. The development of these antibodies occurred as follows: (1) m-tACPA was derived from a hybridoma screen of mice that were immunized with a citrullinated N-terminal H2A peptide; (2) a chimera (ch-tACPA) was generated from m-tACPA that contained the mouse variable domains and human constant domains; (3) a humanized version (hz-tACPA) was generated from ch-tACPA by CDR grafting and germlining toward a human germline; finally (4) an isomerization site was removed from the light chain CDR1, which resulted in a fully optimized development candidate (dc-tACPA) and backup tACPA molecules that are suitable for large-scale production of clinical grade batches, which can be tested in patients. During the course of lead optimization efforts of tACPA, we tested the individual molecules, which all demonstrated NET-inhibiting capacities in vitro, as well as pharmacological activity in the CAIA mouse model of IA. Table 1 provides an overview of the different tACPA molecules that were used in this study.

**Table 1:** Overview of tACPA molecules used in this study

Name	Format	Isotype	Derived from	Features	Target	NET inhibition	Pharmacological activity (mouse models)
h-tACPA	Human	hIgG1/κ	Human scFv RA library	NA	citH2A and citH4	Yes	CAIA
m-tACPA	Mouse	mIgG1/κ	Hybridoma screen	NA	citH2A and citH4	Yes	CAIA, PF, colitis
ch-tACPA	Chimerized	h-mIgG1/κ	m-tACPA	Mouse variable and human constant domains	citH2A and citH4	Yes	CAIA
hz-tACPA	Humanized	hIgG1/κ	ch-tACPA	CDR grafted and germlined	citH2A and citH4	Yes	CAIA, sepsis, CIA
dc-tACPA	Development candidate	hIgG1/κ	hz-tACPA	Isomerization removed in light chain CDR1	citH2A and citH4	Yes	CAIA, peritonitis

*NA* not applicable

We engineered different tACPA molecules that have distinct features. Each time an improved molecule became available, we used it in our experiments. The development of these antibodies occurred step-by-step as follows: (1) h-tACPA was obtained from a human scFv RA library screen. The tACPA target was discovered using h-tACPAs; (2) m-tACPA was derived from a hybridoma screen; (3) ch-tACPA was generated from m-tACPA; (4) hz-tACPA was generated through ch-tACPA optimization; and (5) hz-tACPA optimization finally resulted in dc-tACPA. During the course of lead optimization efforts of tACPA, we tested the individual molecules, which all demonstrated NET-inhibiting capacities in vitro as well as in vivo pharmacological activity in the CAIA mouse model of IA

### The therapeutic effect of tACPA is proven using a preclinical basket approach with various neutrophil-mediated disease models

To demonstrate the therapeutic efficacy of tACPA, we used four different mouse models in our preclinical basket approach, including CAIA, bleomycin-induced pulmonary fibrosis (PF), dextran sulfate sodium (DSS)-induced colitis, and lipopolysaccharide (LPS)-induced sepsis. The mouse models included in the preclinical basket trial approach were selected for several reasons: (1) the presence of target tACPA epitopes; (2) the presence of a similar inflammatory response as that in corresponding human diseases with a high unmet clinical need; (3) a plausible NET-mediated pathology; or (4) a previously described therapeutic response to PAD4 inhibitors.

**Fig. 1:**
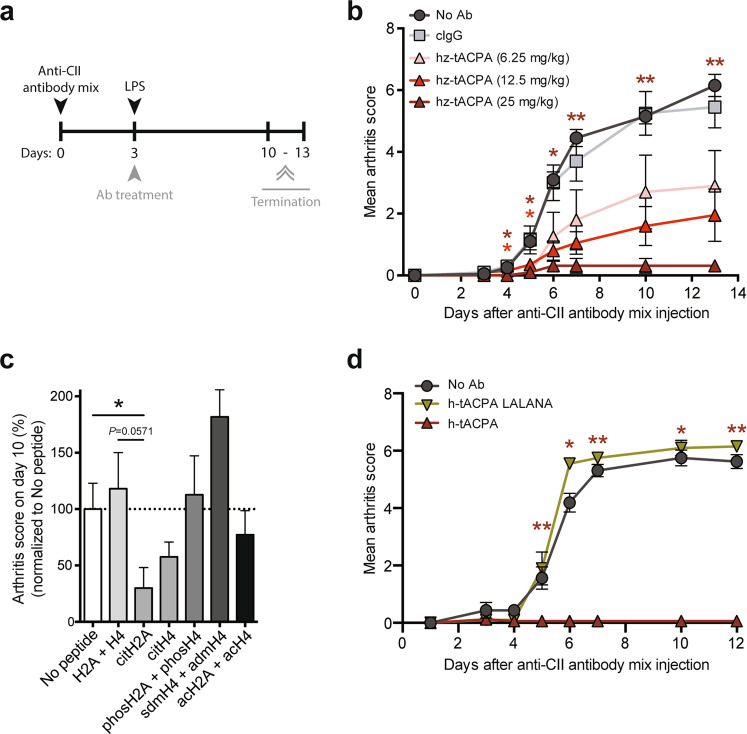
tACPA prevents disease progression in CAIA mice. **a** A schematic overview of the CAIA mouse model of IA. Mice were injected with the anti-CII antibody mixture, followed by LPS at day 3, to induce acute arthritis. Antibody (Ab) treatment started directly after LPS injection by administration of hz-tACPA (6.25, 12.5, or 25 mg/kg), cIgG (25 mg/kg) or PBS (No Ab). Fifty mg/kg antibody was used in the experiment in which h-tACPA LALANA was compared with h-tACPA and cIgG. The mice were terminated on days 10–13. **b** The mean arthritis score (MAS) of CAIA mice was evaluated for 13 days (*n* = 5 mice per group; cIgG was used to calculate significant differences). **c** Prior to anti-CII antibody mixture and LPS injection, the mice were immunized with different posttranslationally modified H2A and H4 peptides (Supplementary Table 2), including unmodified H2A and H4 (H2A + H4), citrullinated H2A (citH2A), citrullinated H4 (citH4), phosphorylated H2A and H4 (phosH2A + phosH4), acetylated H2A and H4 (acH2A + acH4), and symmetric and asymmetric methylated H4 (sdmH4 + admH4). Non-immunized mice (no peptide) were used as controls. Only mice that developed a specific immune response against these peptides were selected for inclusion in the CAIA experiment (Supplementary Table 3). The MAS of immunized CAIA mice was evaluated at day 10 (*n* = 3–9 mice per group; the mean of the ‘No peptide’ group was set at 100%, and individual percentages were calculated). **d** The MAS of CAIA mice was evaluated for 12 days (*n* = 4–5 mice per group; h-tACPA and h-tACPA LALANA were used to calculate significant differences). The results are presented as the means ± SEM. *P < 0.05, **P < 0.01, using two-tailed Mann–Whitney statistical test

We performed a prophylactic dose-response hz-tACPA treatment regimen in a CAIA mouse model of IA (Fig. 1a). The injection of an anti-collagen type II (anti-CII) antibody mixture followed by LPS on day 3 induced severe inflammation in all paws, which was quantified by macroscopic assessment of the mean arthritis score (MAS). Supplementary Table 1 shows the scoring system for macroscopic signs of inflammation. Administration of 6.25 and 12.5 mg/kg hz-tACPA reduced the MAS at day 13 by 47% and 64%, respectively, compared with that of the isotype control antibody (cIgG). More importantly, 25 mg/kg hz-tACPA almost completely prevented disease development (94% reduction at day 13; Fig. 1b). Even when mixed together with a human ACPA, tACPA maintained its therapeutic activity (Supplementary Fig. 2).

To determine the potency of citH2A and citH4 as therapeutic targets compared with that of other histone post-translational modifications (PTMs), we immunized DBA/J1 mice with different N-terminally modified H2A and H4 peptides (Supplementary Table 2). Only mice that developed a clear immune response against these peptides were selected for inclusion in the CAIA experiment (Supplementary Table 3). After injection of the anti-CII antibody mixture on day 0, followed by an LPS injection on day 3, we determined a reduced MAS on day 10 only in mice with serum IgG against the citH2A peptide and, to a lesser extent, in mice that had an IgG response against the citH4 peptide compared with that of nonimmunized mice (No peptide). Interestingly, mice that had an immune response against N-terminal histone peptides without PTMs (H2A + H4), phosphorylated histone peptides (phosH2A + phosH4), or acetylated histone peptides (acH2A + acH4) were not protected against anti-CII antibody-induced inflammation in their paws (Fig. 1c). Moreover, mice with an IgG immune response against symmetric and asymmetric methylated histone peptides (sdmH4 + admH4) showed a marked increase in macroscopic paw inflammation on day 10.

To study whether the Fc domain of tACPA is important for its therapeutic ability, we created an h-tACPA molecule with complete loss of Fcγ receptor (FcγR) binding affinity by amino acid substitutions (L234A, L235A, and N297A) in the Fc domain of the antibody (referred to as h-tACPA LALANA). Administration of 50 mg/kg h-tACPA LALANA in a CAIA mouse model of IA did not show any therapeutic effect compared with that of cIgG, whereas wild-type h-tACPA again completely prevented disease development (Fig. 1d). Of note, LALANA-mutated antibodies contained similar pharmacokinetics in mice and rats compared with those of wild-type antibodies.^27–29^ Together, these results demonstrate that specific N-terminal citrullinated epitopes on H2A and H4 are targets for therapeutic intervention, whereas antibodies against other N-terminal histone PTMs or histones without PTMs do not have a therapeutic effect or even exacerbate the pathology. Furthermore, the Fc domain of tACPA is essential for its therapeutic effect, suggesting a role of FcγRs in this process.

**Fig. 2:**
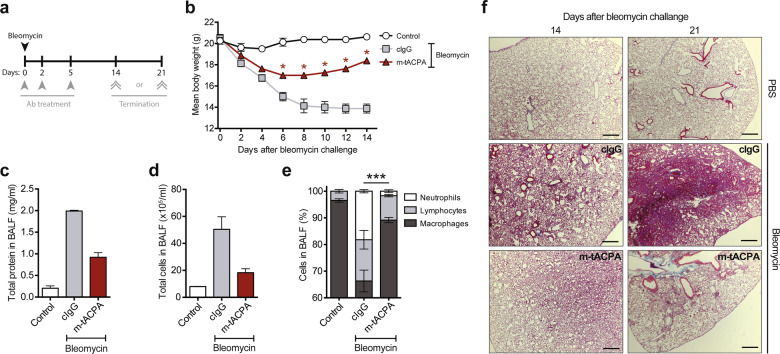
tACPA prevents lung fibrosis in bleomycin-induced PF mice. **a** A schematic overview of the bleomycin-induced PF mouse model. Mice were challenged with PBS (Control) or 2.5 U/kg bleomycin to induce acute PF. Antibody (Ab) treatment was performed with three injections of 50 mg/kg cIgG or m-tACPA at days 0, 2, and 5. The mice were terminated at day 14 or 21. **b** The body weights of bleomycin-challenged mice was evaluated over time (*n* = 4 mice per group). BALF was collected at day 14 and analyzed for the presence of **c** total protein and **d** total cells (*n* = 2; other lungs were used for H&E staining). **e** Cell composition in BALF was determined as described elsewhere^89^ (*n* = 2; neutrophils were used to calculate significant differences). **f** Representative images showing H&E staining of the lungs of cIgG- or m-tACPA-treated mice that were challenged with bleomycin or PBS at 14 and 21 days. Scale bars: 100 µm. The results are presented as the means ± SEM. *P < 0.05, ***P < 0.001, using two-tailed Mann–Whitney statistical test (b; cIgG was used to calculate significant differences) or two-way analysis of variance (ANOVA) with Tukey’s multiple comparisons test (**e**)

Next, we used the bleomycin-induced PF mouse model to study the disease-modifying effect of m-tACPA in the formation of fibrotic lesions in the lung (Fig. 2a). Bleomycin-induced illness in mice was determined by rapid weight loss. The body weights of cIgG-treated mice were severely reduced at day 14 compared with those at day 0 (31% reduction; Fig. 2b). Mice that were treated with m-tACPA showed a slight decline in body weights during the first days after bleomycin challenge. However, their body weights steadily rose again from day 8 onwards, leading to significantly increased body weights at day 14 compared with those of cIgG-treated mice. Evidence of PF was established by analyzing proteins and cells in bronchoalveolar lavage fluid (BALF) 14 days after bleomycin challenge. Treatment with m-tACPA reduced the amount of proteins (Fig. 2c) and cells (Fig. 2d) in BALF compared with those of cIgG treatment. Intriguingly, the composition of neutrophils, lymphocytes, and macrophages in the BALF of m-tACPA-treated mice reflected near physiological cell counts when compared with those of PBS-challenged mice (Control). BALF from m-tACPA-treated mice contained 1.7% neutrophils, whereas cIgG-treated mice had a neutrophil influx of 18.4% (Fig. 2e). PF in the lungs was visualized with hematoxylin and eosin (H&E) staining and was present in cIgG-treated mice at days 14 and 21 after bleomycin challenge, while m-tACPA-treated mice lacked PF (Fig. 2f). These data suggest that tACPA reduces neutrophil-driven inflammation and decreases fibrosis in the lung. Due to the lengthy timeframe of this experiment, it is impossible to determine whether the therapeutic effect of tACPA occurred because of direct inhibition of neutrophil influx or the mitigation of inflammatory triggers. This issue is investigated elsewhere in this study using a pristane-induced peritonitis mouse model.

**Fig. 3:**
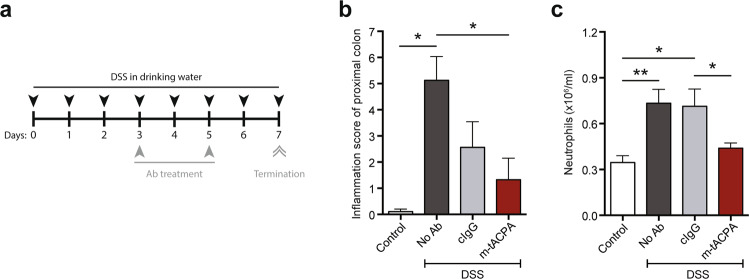
tACPA prevents inflammation and tissue injury in DSS-induced colitis mice. **a** A schematic overview of the DSS-induced colitis mouse model of IBD. Mice were challenged for 7 days with normal drinking water (Control) or drinking water containing 5% (w/v) DSS and were injected with PBS (No Ab), 50 mg/kg cIgG, or m-tACPA at days 3 and 5. The mice were terminated at day 7. **b** The inflammation score of the proximal colon was analyzed at day 7 (Supplementary Table 4). **c** The neutrophil count in blood was evaluated at day 7. The results are presented as the means ± SEM (*n* = 5–8 per group for (**b**) and (**c**)). *P < 0.05, **P < 0.01, using two-tailed Mann–Whitney statistical test (**b**) or unpaired two-tailed Student’s *t* test (**c**)

DSS-induced colitis in mice has many similarities with human inflammatory bowel disease (IBD), including neutrophil-mediated pathogenesis of the colonic mucosa.^30,31^ To determine the therapeutic effect of m-tACPA in the IBD model, mice were exposed to DSS in their drinking water for the entire time span of the experiment, and m-tACPA, cIgG, or PBS (No Ab) was injected in a therapeutic fashion on days 3 and 5 (Fig. 3a). Although the mice did not show illness or lost weight during DSS exposure for 7 days (Supplementary Fig. 3), the development of colitis occurred. After histological analysis of the gut, we found inflammation and tissue damage in the proximal colon of PBS- and cIgG-treated animals, whereas m-tACPA treatment resulted in a significantly decreased inflammation score compared with that of PBS-treated mice (Fig. 3b). Supplementary Table 4 shows the scoring system for tissue damage in the proximal colon of DSS-induced colitis mice. In this model, we also found a strong neutrophil effect in m-tACPA-treated mice, restoring circulating blood neutrophils back to physiological levels (similar to that of the control), while neutrophil levels in PBS- and cIgG-treated animals were significantly increased (Fig. 3c). These data, together with the effect of m-tACPA on BALF neutrophil levels in the bleomycin-induced PF model, provide additional evidence that tACPA could be of therapeutic value in inflammatory diseases beyond RA.

**Fig. 4:**
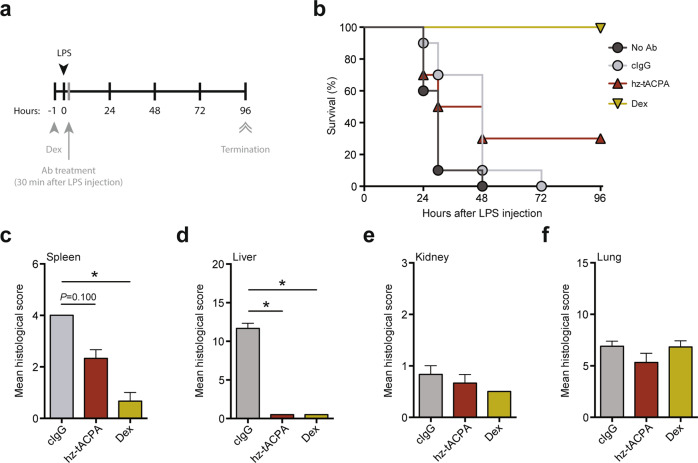
tACPA enhances survival and prevents tissue damage in LPS-induced sepsis mice. **a** A schematic overview of the LPS-induced sepsis mouse model. Mice were injected with 10 mg/kg LPS, and 30 min later, PBS (No Ab), 50 mg/kg cIgG, or hz-tACPA was administered. Dexamethasone (Dex; 10 mg/kg) was administered 1 h prior to LPS challenge. The mice were terminated at 96 h after LPS injection. **b** Survival was monitored for 96 h (*n* = 10 mice per group). Histological analysis was performed on the **c** spleen, **d** liver, **e** kidney, and **f** lung (*n* = 3; Supplementary Table 5). The results are presented as the means ± SEM. *P < 0.05, using one-way ANOVA with post hoc Dunn’s test for Kruskal–Wallis multiple comparisons test

Overwhelming neutrophil-mediated inflammation, secondary to infection, has been demonstrated in severe human sepsis,^32^ whereas a beneficial effect of the PAD inhibitor Cl-amidine has been demonstrated in an LPS-induced mouse model of sepsis.^33^ Therefore, we decided to include an LPS-induced sepsis mouse model in our preclinical basket approach. Furthermore, the pathology present in this LPS-induced sepsis model is similar to that in human vasculitis and acute respiratory distress syndrome, making it also a surrogate model for these diseases.^13,34,35^ To investigate the efficacy of hz-tACPA in this model of acute inflammation, mice were intraperitoneally injected with LPS and treated in a therapeutic setting (Fig. 4a). LPS administration in combination with cIgG treatment resulted in a 100% mortality rate within 72 h, whereas hz-tACPA treatment enhanced survival rates up to 30% within 96 h (Fig. 4b). Interestingly, mice that received hz-tACPA were healthy without typical signs of endotoxemia until their death, while PBS-treated (No Ab) and cIgG-treated mice became severely ill (piloerection, little activity, and not eating or drinking) immediately after LPS injection. Dexamethasone (Dex) treatment was used in a prophylactic setting 1 h prior to LPS injection and induced 100% survival in this animal model, thus serving as a positive control for treatment. To obtain further insight into the protective effect of hz-tACPA, histological analysis of the spleen, liver, kidney, and lung was performed according to the scoring system for tissue damage of these organs (Supplementary Table 5). Treatment with hz-tACPA in LPS-induced sepsis mice showed a reduction in spleen (Fig. 4c) and liver damage (Fig. 4d) by ~50% and ~95%, respectively, compared with those of cIgG-treated mice. Kidney (Fig. 4e) and lung damage (Fig. 4f) were not affected by hz-tACPA treatment. However, it is noteworthy that Dex treatment did not reduce kidney or lung damage either, which was similar to the effects of hz-tACPA treatment.

Together, these data indicate the in vivo therapeutic efficacy of tACPA in several murine models that are associated with neutrophil-mediated pathogenesis. The therapeutic effects of tACPA in the above-described mouse models, together with the fact that tACPA binds citrullinated histone epitopes^26^ that are known to be necessary for NET formation, prompted us to investigate the NET-inhibiting capacities of tACPA.

### tACPA inhibits NET formation in vivo and does not influence neutrophil recruitment

Citrullination of histones is associated with the formation of NETs^21^ and has been detected in tissue and joints of patients and murine models of various diseases.^17,33^ To investigate whether tACPA interferes with NET formation in vivo, we used a pristane-induced mouse model of peritoneal cell influx, which has previously been described by Kienhöfer and colleagues.^36^ In contrast to the previously described mouse models, this model is highly suitable to studying the direct in vivo NET-inhibitory effect of tACPA, since pristane induces a fast and robust inflammatory cell influx, including neutrophils that form NETs, which can be easily isolated from the peritoneum.

**Fig. 5:**
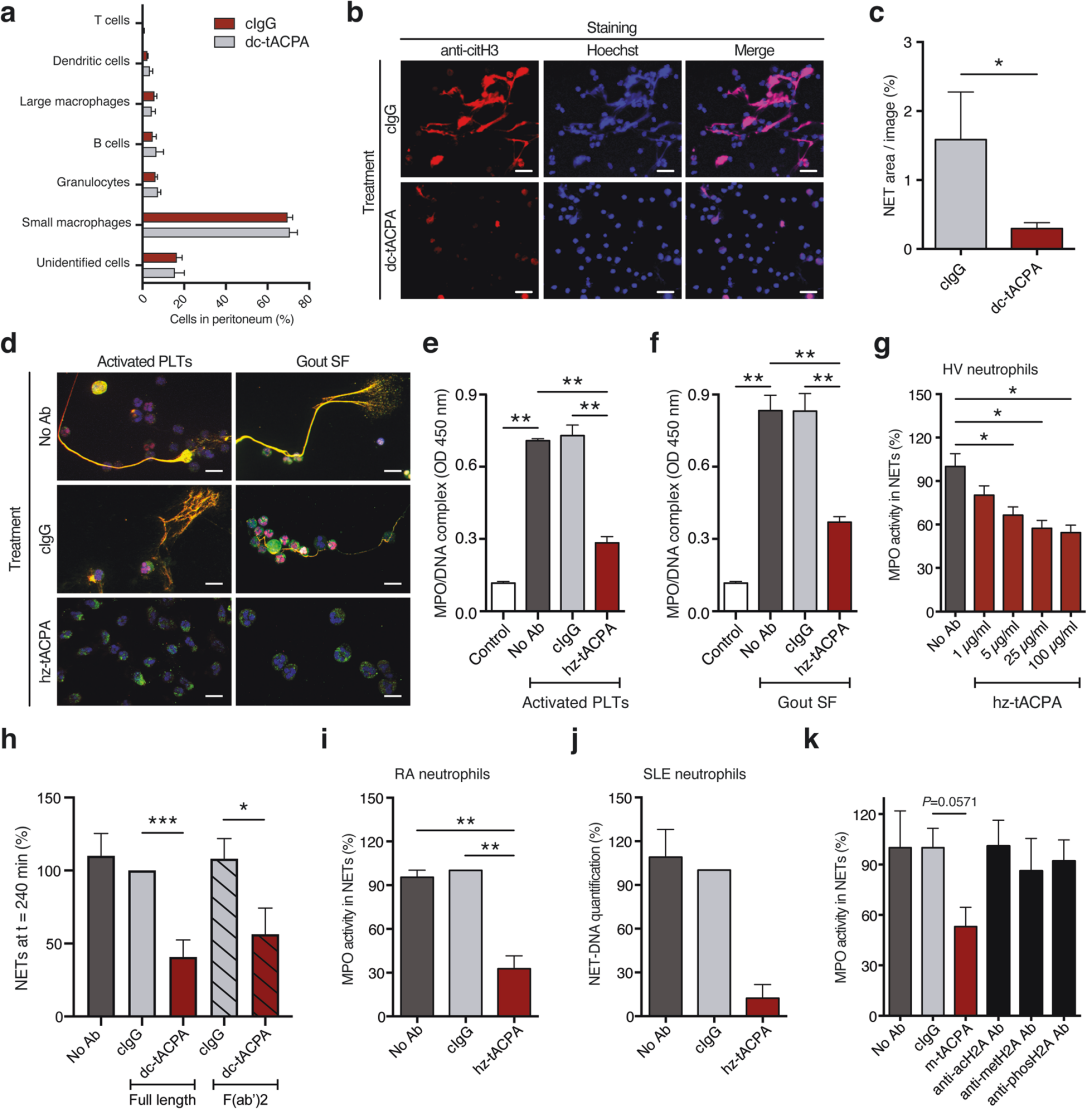
tACPA inhibits NET formation in vivo and in vitro. To induce inflammation in the peritoneum, pristane was injected, followed by cIgG or dc-tACPA. **a** The composition of peritoneal cell infiltrates was analyzed after 24 h (*n* = 7–8). **b** Representative images showing NET formation in the peritoneum of mice treated with cIgG or dc-tACPA. NETs were stained with Hoechst (blue) and anti-citH3 antibody (red). Scale bars: 50 µm. **c** Quantification of NETs (colocalization of citH3 and Hoechst; *n* = 10). **d** Representative images showing in vitro human NET release induced by activated PLTs or gout SF without antibody (No Ab) or in the presence of cIgG or hz-tACPA. Scale bars: 20 µm, bottom right image: 10 µm. NETs were stained with DAPI (blue), anti-citH3 antibody (red), and anti-NE antibody (green). MPO/DNA complexes were measured in harvested NETs upon stimulation with activated **e** PLTs or **f** gout SF (*n* = 5). **g** The activity of MPO present in NETs of neutrophils from HVs was measured after A23187 stimulation in the presence of different concentrations of hz-tACPA (*n* = 4). **h** The percentage of NETs at *t* = 240 min in A23187-stimulated neutrophils from HVs that were treated with either full-length dc-tACPA or dc-tACPA F(ab′)2 antibody fragments was quantified (*n* = 8). MPO activity in NETs or NET-DNA was quantified in A23187-stimulated neutrophils from **i** RA and **j** SLE patients (*n* = 5 and *n* = 3, respectively) in the absence or presence of cIgG or hz-tACPA. **k** MPO activity in human A23187-induced NETs without antibodies or with cIgG, m-tACPA, antibodies against acetylated H2A (anti-acH2A), methylated H2A (anti-metH2A), or phosphorylated H2A (anti-phosH2A) was measured (*n* = 4). To determine the percentage of DNA and MPO activity in NETs, the mean of the No Ab group was set at 100%, and individual percentages were calculated (**g** and **k**). In (**i**) and (**j**), the mean of the cIgG group was set at 100%. The results are presented as the means ± SEM. *P < 0.05, **P < 0.01, ***P < 0.001 using two-tailed Mann–Whitney statistical test

Twenty-four hours after intraperitoneal (i.p.) treatment with pristane, followed by tACPA or cIgG, inflammatory cells were isolated from the peritoneum and prepared for FACS analysis and immunofluorescence (IF) microscopy to analyze the cellular composition and NET release, respectively. Importantly, the cellular composition of the peritoneal infiltrates was similar in cIgG- and dc-tACPA-treated mice (Fig. 5a), showing that tACPA does not influence the migratory capacity of inflammatory cells. Moreover, we observed a decrease in NET filaments containing DNA and citH3 in peritoneal cells from dc-tACPA-treated mice compared with those of cIgG-treated mice (Fig. 5b). Quantification of NETs (colocalization of citH3 and Hoechst) confirmed this observation (Fig. 5c). We further demonstrated the ability of dc-tACPA to inhibit mouse NET formation in an in vitro experiment using A23187-treated mouse bone marrow (BM)-derived neutrophils (Supplementary Fig. 4). Together, these data indicate that tACPA inhibits NET release in mice.

### tACPA inhibits human NET formation in response to physiological stimuli

To investigate whether tACPA inhibits NET formation induced by physiologically relevant human disease-related stimuli for vasculitis and gout, human neutrophils from healthy volunteers (HVs) were stimulated with activated human platelets (PLTs) or synovial fluid (SF) from gout patients to form NETs. NET structures, including DNA, NE, and citH3, were observed (Fig. 5d). Neutrophils that were treated with hz-tACPA during stimulation with activated human PLTs or gout SF showed decreased NET release compared with that of untreated (No Ab) or cIgG-treated neutrophils (Fig. 5d and Supplementary Fig. 5a, b). The IF microscopy data were confirmed by measuring MPO/DNA complexes in the supernatant of neutrophils that were stimulated with activated human PLTs (Fig. 5e) and gout SF (Fig. 5f). The NET-inhibiting properties of hz-tACPA were further investigated by triggering neutrophils with the calcium ionophore A23187, a robust NET inducer that we used for routine experiments. Neutrophils from HVs released NETs upon A23187 stimulation, which were inhibited with hz-tACPA in a dose-dependent manner (Fig. 5g). In addition, dc-tACPA F(ab′)2 antibody fragments also inhibited NET release, which demonstrates that the capacity of tACPA to inhibit NETs in vitro is FcγR independent (Fig. 5h). Furthermore, A23187-induced NET release in neutrophils from RA and SLE patients was reduced when the cells were treated with hz-tACPA (Fig. 5i, j, and Supplementary Fig. 6), which proves the NET-inhibiting capacity of tACPA in neutrophils from patients with autoimmune diseases. Subsequently, The NET-inhibiting capacities of tACPA were tested in the presence of ACPA. For this purpose, anti-cyclic citrullinated peptide positive (CCP+) RA SF was used as an additional ACPA-containing physiological NET inducer. ACPA-containing RA SF induced NET formation, and hz-tACPA inhibited this effect (Supplementary Fig. 5c). To determine the involvement of citH2A and citH4 as targets for NET inhibition compared with other histone PTMs, we used anti-acH2A, anti-methylated H2A (metH2A), and anti-phosH2A to inhibit NET release. Remarkably, only m-tACPA inhibited A23187-induced NET release, while anti-acH2A, anti-metH2A, and anti-phosH2A antibodies did not diminish NET release (Fig. 5k). These observations demonstrate that tACPA inhibits NET release upon in vitro stimulation with different physiological triggers in neutrophils from human HVs, as well as in RA and SLE patients, and that citH2A and citH4 are specific targets for the inhibition of NET formation, whereas other histone PTMs are not.

### tACPA-opsonized NETs are phagocytosed by macrophages in vivo

**Fig. 6:**
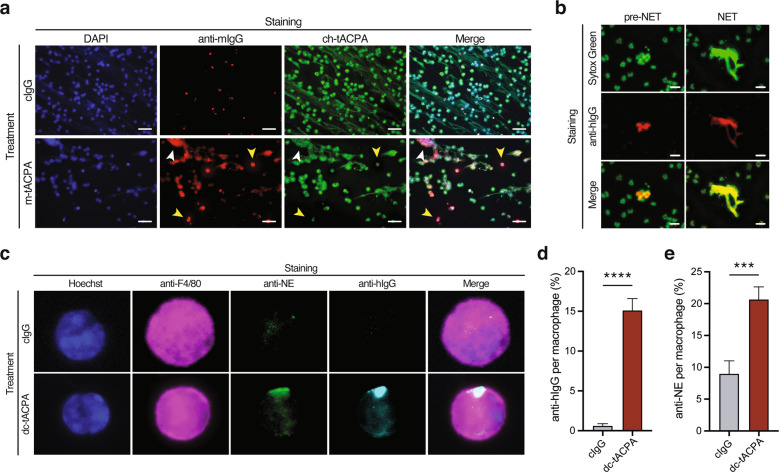
tACPA binds to (pre-)NETs, which are taken up by macrophages in vivo. **a** Representative images of tACPA binding in A23187-induced NET release in HV neutrophils. NETs were stained with DAPI (blue), anti-mIgG antibody (red), and ch-tACPA (green). Binding of m-tACPA and ch-tACPA to pre-NETs and extracellular NETs is indicated with yellow and white arrowheads, respectively. Scale bars: 50 µm. To induce peritoneal cell infiltration and NET formation, pristane was injected into the peritoneum of mice with 1 or 2 i.p. injections of 50 mg/kg dc-tACPA at 0 h (**b**) or at 0 and 12 h (**c**, **d** and **e**). After 24 h, inflammatory cells were harvested from the peritoneum and analyzed. **b** Representative images showing dc-tACPA binding to pre-NETs and NETs from the peritoneum of mice with pristane-induced peritonitis. NETs and dc-tACPA were stained with Sytox Green and anti-human IgG (red), respectively. Scale bars: 25 µm. **c** Representative images of tACPA-opsonized NETs, which have been phagocytosed by macrophages. NETs containing NE in combination with hIgG (tACPA) were present in macrophages from animals that had been treated with dc-tACPA only. Hoechst (blue), the macrophage marker F4/80 (magenta), anti-NE (green) and anti-hIgG (cyan). Scale bars: 10 µm. **d**, **e** Quantification of phagocytosed hIgG (% anti-hIgG per macrophage image) and neutrophil elastase (% anti-NE per macrophage image) (*n* = 58–176 macrophages from three mice). The results are presented as the means ± SEM. ***P < 0.001, ****P < 0.0001 using two-way ANOVA with Dunnett’s multiple comparisons test

In addition to inhibiting the process of NET formation, the anti-inflammatory effect of tACPA may also be a result of NET clearance from circulation and affected tissues. The binding of tACPA to existing NETs could be the first step toward clearance of NETs by macrophages. To study this hypothesis, human neutrophils were stimulated with A23187 to induce NET release in the presence of cIgG or m-tACPA, and after washing and fixation, the presence of m-tACPA was visualized using a secondary anti-mouse IgG-Alexa594 antibody. NETs were targeted by m-tACPA (Fig. 6a; white arrowhead in the anti-mIgG lower panel). Interestingly, m-tACPA also bound to neutrophils that were in an early phase of NET release (pre-NET), again proving the NET-inhibiting effect of tACPA (Fig. 6a; yellow arrowheads in the anti-mIgG lower panel). We defined pre-NETs as neutrophils with an amorphous decondensed nuclear structure containing citrullinated chromatin that still appeared intracellularly, having a collapsed nuclear membrane and most likely a porous/punctured cell membrane. Following fixation, costaining with ch-tACPA (recognizes identical epitopes as m-tACPA but is a chimeric antibody) clearly showed binding to pre-NETs as well as expelled NETs (Fig. 6a; yellow and white arrowheads, respectively, in both the ch-tACPA and Merge lower panels). The binding of tACPA to human NETs was confirmed in an independent sandwich ELISA experiment, in which hz-tACPA was used to capture NETs, while an anti-MPO antibody was used to detect these NETs (Supplementary Fig. 7). To further investigate the binding of tACPA to mouse (pre-)NETs, we used A23187-treated BM-derived neutrophils, as well as the pristane-induced peritonitis mouse model. As expected, we observed dc-tACPA (anti-hIgG) binding to (pre-)NETs in vitro (Supplementary Fig. 8), as well as to pre-NETs and expelled NETs in vivo (Fig. 6b; left and right panels, respectively). Interestingly, among the peritoneal pristane-induced cellular infiltrates, we observed F4/80-positive macrophages containing phagocytosed dc-tACPA (citH2A and citH4) in combination with enhanced amounts of the NET component neutrophil elastase (Fig. 6c), which is quantified and shown in Fig. 6d, e, respectively.

Together, these data indicate that tACPA binds to expelled human and mouse NETs both in vitro and in vivo, which is the first step toward macrophage-induced NET uptake.

### tACPA prevents NET-induced cartilage and bone erosion and disease progression in a CIA mouse model of IA

**Fig. 7:**
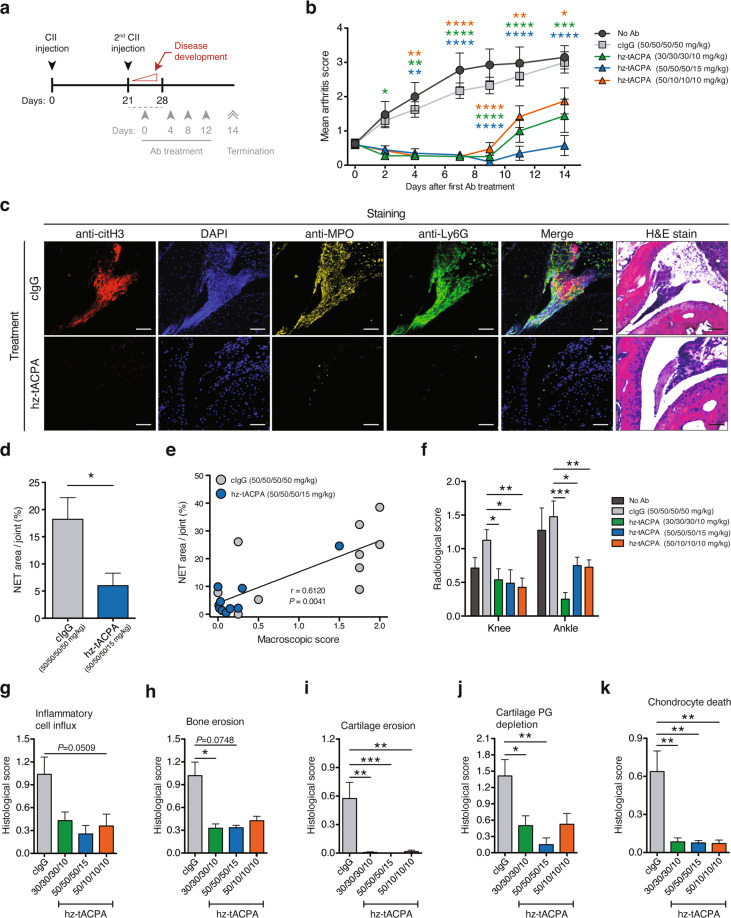
tACPA prevents NET-mediated tissue damage and disease progression in chronic CIA mice. **a** A schematic overview of the CIA mouse model of IA. To induce chronic IA, mice were injected twice (days 0 and 21) with CII. Therapeutic treatment started after onset of the disease (between days 21 and 28), when the MAS was ≥0.75, and included four injections (4-day intervals) with tapered dosing regimens of cIgG (50/50/50/50 mg/kg) or hz-tACPA (30/30/30/10, 50/50/50/15 or 50/10/10/10 mg/kg). The mice were terminated 14 days after the start of treatment. **b** The MAS of CIA mice was evaluated for 14 days (*n* = 10 mice per group). **c** Representative immunofluorescence and H&E images of NET release in joints of right hind paws showing citH3 (red), DAPI (blue), MPO (yellow), and Ly6G (green). Scale bars: 100 µm. **d** Quantification of NETs (colocalization of citH3 and MPO) in the tibiotarsal, proximal intertarsal, distal intertarsal, and tarsometatarsal joints of the right hind paws of mice (*n* = 10). **e** Significant correlation of macroscopic score (paw swelling) and NETs per joint. **f** Bone damage to the right and left hind knees and ankles was analyzed by X-ray at day 14 after the first antibody injection (*n* = 10). H&E and SO staining of joints from right and left ankles showing **g** inflammatory cell influx, **h** bone erosion, **i** cartilage erosion, **j** cartilage PG depletion, and **k** chondrocyte death at day 14 after the first antibody injection (*n* = 16–20 mouse ankles). The results are presented as the means ± SEM. *P < 0.05, **P < 0.01, ***P < 0.001, ****P < 0.0001 using two-way ANOVA with Dunnett’s multiple comparisons test (**b**; cIgG was used to calculate significant differences), unpaired two-tailed Student’s *t* test (**d**), two-tailed Mann–Whitney statistical test (**f**–**k**), or Spearman *r* test (**e**)

To investigate the efficacy of tACPA in NET-induced tissue damage, we used different tapered tACPA strategies in a chronic collagen-induced arthritis (CIA) mouse model of IA. Two injections of CII at days 0 and 21 induced inflammation in the hind paws, followed by hz-tACPA or cIgG treatment (Fig. 7a). Antibody treatment was started between days 21 and 28 when an MAS of ≥0.75 was reached. Therapeutic administration with four repeated i.v. injections 4 days apart (Fig. 7a; gray arrowheads) with the indicated doses of hz-tACPA (50/10/10/10, 30/30/30/10, and 50/50/50/15 mg/kg) reduced the MAS at day 14 by 38%, 52%, and 81%, respectively, compared with that of treatment with 50/50/50/50 mg/kg cIgG (Fig. 7b). Notably, all hz-tACPA administrations prevented disease development during the first 8 days, after which the MAS started to rise, possibly due to the development of anti-drug antibodies in these mice. Only treatment with 50/50/50/15 mg/kg hz-tACPA completely stabilized the disease for a total of 14 days, without exceeding an MAS of 0.75.

We then investigated the presence of NETs in the paws of CIA mice that received 50/50/50/15 mg/kg hz-tACPA or 50/50/50/50 mg/kg cIgG. Neutrophils (Ly6G), MPO, and citH3 were observed in cIgG-treated animals, whereas these markers were nearly absent in tACPA-treated mice (Fig. 7c). Quantification of NETs (colocalization of citH3 and MPO) was performed by analyzing multiple joints in the right hind paw of each animal, including the tibiotarsal joint, the proximal intertarsal joint, the distal intertarsal joint, and the tarsometatarsal joint. A decrease in NETs (Fig. 7d) was observed in the joints of tACPA-treated mice compared with that of cIgG-treated mice. We found that the amount of NETs in the joint significantly correlated with macroscopic paw swelling (Fig. 7e; *r* = 0.6120, *P* = 0.0041).

NETs have been characterized in inflamed joints of RA patients and eventually cause progressive tissue and bone damage.^17^ To further study the effect of tACPA-induced NET inhibition on bone damage, we performed X-ray analysis of the knees and ankles of all hind paws from the above-described hz-tACPA and cIgG treatment groups. Supplementary Fig. 9 shows example X-ray images of knees and ankles from 50/50/50/15 mg/kg hz-tACPA- and 50/50/50/50 mg/kg cIgG-treated mice, in which bone erosion (white arrowheads) was only observed in the cIgG-treated mice. Consistent with the observed MAS, all hz-tACPA treatments suppressed bone damage in both ankles and knees (Fig. 7f). To obtain further insight into the protective effect of tACPA, histological analysis of ankle joints was performed using H&E and safranin O (SO) staining. Treatment with hz-tACPA inhibited inflammatory cell influx compared with that of cIgG-treated mice (Fig. 7g and Supplementary Fig. 10; asterisks in the upper panel). Furthermore, hz-tACPA significantly reduced bone and cartilage erosion, cartilage proteoglycan depletion and chondrocyte death compared with those of cIgG-treated mice (Fig. 7h–k and Supplementary Fig. 10; lower panel). Together, these data indicate that tACPA treatment results in eradication of NETs in inflamed tissue in vivo, thereby preventing the symptoms of arthritis, including severe bone and tissue damage in joints.

## Discussion

Here, we showed that tACPAs, antibodies that specifically recognize citH2A and citH4, have therapeutic efficacy in murine models of neutrophil-mediated inflammatory diseases, including IA, PF, IBD, and sepsis. Exacerbation and progression of the disease was prevented or attenuated in all models upon tACPA treatment. Furthermore, we demonstrated that tACPA inhibits human and mouse NET formation induced by distinct physiological stimuli both in vivo and in vitro and that tACPA binds to pre-NETs and expelled NETs, which are then taken up by macrophages. Finally, we showed in a chronic CIA mouse model of IA that tACPA treatment prevents NET release in hind paw tissue. Our findings suggest that the therapeutic effect of tACPA in neutrophil-mediated inflammatory diseases acts through NET inhibition and potentially through initiating the uptake and digestion of pre-NETs and NETs by macrophages, thereby eliminating the noxious triggers that lead to chronic inflammation and tissue damage.

ACPAs are autoantibodies that are present in the majority of RA patients and are used as powerful diagnostic and prognostic tools. The presence of ACPAs and their binding to citrullinated targets within the inflamed synovium is associated with disease severity and enhanced tissue injury in RA patients and murine models of RA.^37–41^ In addition, the generation of ACPAs in the early development phases of RA has a strong predictive value for the progression to full-blown disease.^42,43^ However, we previously demonstrated that a small subset of ACPAs, derived from single chain variable fragment (scFv) libraries containing the immune repertoire of RA patients,^44^ exhibit strong therapeutic activity in CAIA and CIA mouse models of IA.^26^ Here, we designate these therapeutic ACPAs as tACPAs. Thus, ACPAs and tACPAs contain opposite properties, such as the induction of either a pro- or anti-inflammatory response, which could be due to different characteristics, including antibody isotype, their glycosylation profile,^45^ their prevalence (reviewed by Seeling et al.^46^ and Silverman et al.^47^), the epitope to which they bind, as well as their cross-reactivity with acetylated or homo-citrullinated/carbamylated proteins.^48–50^ It is plausible that tACPA molecules are extremely rare, low in copy number, and overwhelmed by pathological ACPAs in RA patients, overruling the therapeutic capacities of tACPAs. In SLE, for example, only a small subset of patients has an anti-citH4 autoantibody response, which indicates that these antibodies are rare, and pathological antibodies prevail in this disease.^51^

There is growing evidence of the pathological role of NETs in the development and progression of multiple acute and chronic inflammatory diseases.^5–9,52^ First, NETs act as a source of autoantigens that break immunological tolerance in autoimmune diseases, including RA, SLE, APS, and ANCA-associated vasculitis.^53^ Second, the cytotoxicity of NETs results in local inflammation that leads to tissue damage, organ failure and severe morbidity. Recent data also indicate that NETs play a central role in cancer-initiating tumor progression, metastatic spread, and cancer-associated thrombosis.^54–56^ Together, this suggests that the applicability of NET-targeted therapy is broad. Although the number of reported NET-inhibitory agents has increased since the discovery of NETs, the development of therapeutic drug candidates that target NET formation is still in its infancy.^5,8,57^ To date, genetic or pharmacological abrogation of PAD4 activity in vivo is the only NET-inhibitory approach that has resulted in clear beneficial effects in various murine disease models, including wound healing,^11^ thrombosis^58,59^ atherosclerosis,^22^ sepsis-induced endotoxic shock,^60,61^ colitis,^62^ ischemia/reperfusion-related tissue injury,^63,64^ fibrosis,^65^ IA,^66^ and SLE.^24^ PAD4 has become a therapeutic target in multiple disease models; however, inhibition of PAD4 activity using small molecule drugs comes with a cost. PAD4 expression is not restricted to neutrophils, and its presence has been demonstrated in other hematopoietic cells, in which it citrullinates histones^67^ and transcription factors^68^ to regulate gene expression^69^ and stem cell differentiation.^68^ Moreover, Zhou et al. recently described a direct link between PAD4 and oxidative burst in human neutrophils, showing reduced oxidase activation and bacterial killing upon PAD4 inhibition.^70^ This indicates that targeting PAD4 activity may result in serious side effects. Another protein important in the formation of NETs is Gasdermin D (GSDMD). GSDMD is a pore-forming protein involved in the extrusion of NET DNA and small molecule inhibitors were found to inhibit NET extrusion.^71^ Since GSDMD is expressed in other cell types as well, including CD8+ T cells, it remains to be seen what the therapeutic value would be for both GSDMD and PAD4 inhibitors. Since tACPA interferes with citrullinated histones in NETs just before their release into the extracellular space, but not with the PAD4 enzyme itself, we hypothesize that tACPA does not affect gene transcription or bacterial killing through neutrophils or other cells. Altogether, this suggests that tACPA would be a safer drug without these potential undesired side effects. However, it has been demonstrated that PAD4-driven protein citrullination is not a universal feature of NETs (reviewed in ref. ^72^), which would indicate that tACPA is not suitable for the treatment of diseases that are driven by citrulline void/low NETs.

NETs contain various histone PTMs; however, their presence per se does not make them suitable targets for therapy. Several histone PTMs were found in SLE and RA, including citrullination, acetylation, dimethylation, and phosphorylation,^51,73–75^ as well as specific autoantibodies that target these epitopes.^51,74^ Here, we showed that tACPA inhibits NET formation in vitro, while antibodies against acH2A, metH2A, and phosH2A lack this capacity (Fig. 5k). In addition, immunization of mice with N-terminal citH2A and citH4 peptides, prior to induction of CAIA, protected against aggressive inflammation in their paws, whereas immunization with N-terminal H2A and H4 peptides without any PTMs or containing acetylated or phosphorylated residues had no protective effect in the CAIA model. Moreover, we found that immunization with symmetric and asymmetric methylated H2A and H4 peptides exacerbated inflammation in these mice (Fig. 1c), indicating the importance of targeting the correct histone PTM. Furthermore, Sohn and colleagues showed elevated arthritis levels in CIA mice upon citH2B immunization,^76^ again underscoring the necessity of targeting the correct citrullinated histone. Additionally, we and others have previously demonstrated that ACPAs, which bind to citrullinated fibrinogen or citrullinated cyclic peptide (CFC1), lack therapeutic efficacy in CIA mice.^26,39,41^ Together, these findings emphasize the importance of citH2A and citH4 as NET-specific therapeutic targets.

Here, we demonstrated the therapeutic in vivo efficacy of tACPA using a preclinical basket approach with various murine models of neutrophil-mediated inflammatory diseases. Treatment with tACPA prevented disease symptoms in CAIA and CIA mouse models of RA. NETs are associated with the development of RA^17^ and mediate RA pathogenesis through the activation of synovial fibroblasts in humans.^77^ We provide direct evidence that tACPA diminishes NET deposition in the joints of chronic CIA mice, further preventing tissue damage and disease progression. Additional immunohistochemistry experiments established the binding of tACPA to synovial tissue from human RA patients (Supplementary Fig. 11), indicating the presence of tACPA target in RA patients and the potential for tACPA to become a drug for RA treatment. Since RA-specific markers can be detected long before clinical manifestations,^78^ we suggest that administration of tACPA prior to or immediately at the first signs of RA onset may prevent disease development and pathology. In addition to the therapeutic effect of tACPA in IA models, we also observed a protective effect in an additional set of murine models in which NETs are detrimental for the disease, including sepsis, PF, and IBD. NETs have been detected in biopsies from patients with pediatric IBD, including Crohn’s disease and ulcerative colitis,^79^ as well as in fibrotic interstitial lung tissues, in which NETs have been shown to contribute to PF development by inducing fibroblast differentiation and collagen deposition.^80^ Chrysanthopoulou et al. showed that these fibrotic effects were significantly decreased after degradation of NETs with DNase1, heparin, or a myeloperoxidase inhibitor, indicating the key role of NET-derived components in lung fibroblast differentiation and function.^80^ In addition, the NET component NE contributes to lung fibrosis in the bleomycin-induced PF mouse model.^81^ Furthermore, abundant citH3 and/or NETs cause inflammatory injury by disrupting the microvascular endothelial barrier^82^ and contribute to organ dysfunction by inducing widespread intravascular thrombosis^83^ in mouse models of sepsis. In accordance with these observations, Deng et al showed that neutralizing citH3, using an anti-citH3 monoclonal antibody, attenuates endothelial damage in vitro and improves inflammatory responses as well as survival rates in an in vivo LPS-induced sepsis mouse model.^84^ In humans, the increase in circulating NETs in patients with septic shock is associated with the severity of organ dysfunction and mortality,^85^ suggesting a possible role for tACPA for the treatment of these diseases as well.

**Fig. 8:**
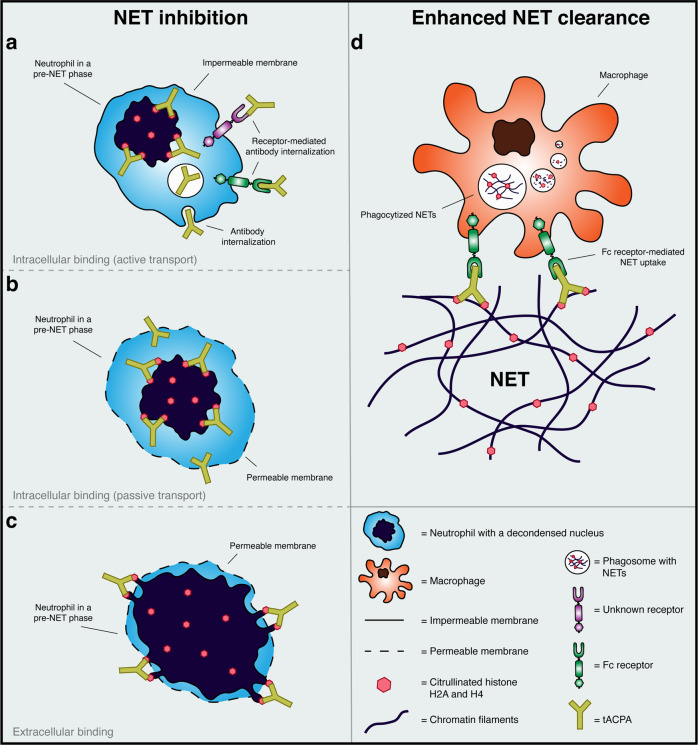
Schematic overview of the plausible mode of action of tACPA. Based on our findings, we hypothesize that tACPA exerts its NET-inhibitory capacity in one of the following ways. tACPA is internalized, either via **a** active or **b** passive transport, and binds to its target, thereby blocking further chromatin expansion. **c** NETs protrude through small holes in the porous plasma membrane, where tACPA binds and prevents final NET expulsion. **d** A pristane-induced peritoneal cell influx mouse model combined with tACPA treatment shows that tACPA binding to NETs might be the first step in enhanced macrophage-induced NET clearance and subsequent protection against continuous inflammation and tissue damage

Citrullination of histones by PAD4 is an essential step in the downstream signaling of NET formation and promotes chromatin decondensation, which is subsequently followed by nuclear membrane disintegration and the release of nuclear material into the cytosol.^21^ During the final stage of NET formation, the plasma membrane is disrupted, and NETs are released into the extracellular environment.^86^ To our knowledge, we are the first to describe an antibody that interferes with NET expulsion to the extracellular space. Based on the intracellular localization of citrullinated histones in the initial phases of NET release, we hypothesize that tACPA exerts its NET-inhibitory capacity in one of the following ways. First, tACPA is internalized, either via active (Fig. 8a) or passive transport (Fig. 8b), and binds to its target, thereby blocking further chromatin expansion. This is not FcγR dependent, since we showed that both full-length dc-tACPA and dc-tACPA F(ab′)2 antibody fragments inhibit A23187-induced NET release in vitro. Second, NETs may protrude through small holes in the porous plasma membrane possibly through Gasdermin D pores,^71^ where tACPA binds extracellularly and prevents final NET expulsion (Fig. 8c). In addition to the direct inhibition of NET release, we also demonstrated that tACPA binds to pre-NETs as well as already expelled NETs (Fig. 6b). Farrera et al. demonstrated that monocyte-derived macrophages engulf NETs and subsequently degrade NETs in their phagosomes without the secretion of proinflammatory cytokines.^87^ Using a pristane-induced peritoneal cell influx mouse model combined with tACPA treatment, we demonstrated that tACPA binding to NETs might be the first step toward enhanced macrophage-induced NET or NETting cell clearance (Fig. 6c–e) and subsequent protection against tissue damage (Fig. 8d). In other words, tACPA has dual activity by directly inhibiting NET formation and by promoting NET and NET component clearance. It is crucial to understand that FcγR engagement by tACPA is not essential for all facets of its multipronged mode of action but is very important for its pharmacological in vivo activity and thus its therapeutic value. Although the NET-inhibitory capacity of tACPA does not depend on FcγRs, tACPA opsonization of pre-NETs and NET clearance by macrophages likely does. Macrophage-mediated phagocytosis is particularly effective when FcγRs are engaged and is initiated when IgG-opsonized particles are encountered (reviewed in ref. ^88^). Considering that non-FcγR-binding-tACPA (h-tACPA LALANA) has no therapeutic effect in the CAIA mouse model of IA (Fig. 1d), we hypothesize that pre-NET and NET clearance by macrophages is an important part of the tACPA mechanism of action. Further investigation is necessary to understand the exact mechanism of action of tACPA at the cellular and systemic levels.

In conclusion, we demonstrated the therapeutic potential of tACPA for the treatment of various acute and chronic disorders in which NETs drive inflammation. The multidimensional capacity of tACPA to prevent NET exposure to the extracellular environment opens up new avenues for the development of therapies for diseases with injurious neutrophilic inflammation.

## Materials and methods

### CAIA mouse model of IA

To induce acute arthritis, 1.6 mg (low dose) or 2.8 mg anti-CII antibody mixture (ModiQuest Research B.V., MQ18.101) was injected i.p. in 10- to 12-week-old male DBA/J1 mice. Three days later, the mice received another i.p. injection containing 25 µg LPS to synchronize the onset of inflammation in the mice. Simultaneously, with LPS administration, the mice received the indicated dose of hz-tACPA, cIgG, or PBS (Fig. 1a); h-tACPA, h-tACPA LALANA or PBS (Fig. 1d); or human ACPA + h-tACPA or only PBS (Supplementary Fig. 2). Typically, inflammation in the front and hind paws became visible beginning 2 days after LPS injection (i.e., day 5). The degree of swelling in the paws was macroscopically scored over a time period of 10–13 days using a scale of 0–2 per paw with a maximal score of 8 per animal: 0 = not inflamed, 1 = mild inflammation, 1.5 = marked inflammation, and 2 = severe inflammation (Supplementary Table 1).

In one particular CAIA experiment, the mice were first immunized with N-terminal H2A and H4 peptides containing different PTMs (Supplementary Table 2). The mice were immunized by subcutaneous injections in the inguinal and axillary regions with emulsions of Freund’s complete adjuvant containing 20 µg peptide conjugated to keyhole limpet hemocyanin (KLH). The mice received 3 i.p. boosters of 30 µg KLH-conjugated peptides at 3-week intervals. Ten days after the last booster, antibody titers were assessed in sera (1:100 diluted in PBS) by ELISA using peptides containing or lacking the particular PTM. Mice that had a specific immune response against the particular PTM but lacked a response against the peptide without the PTM were included in the CAIA experiment (Supplementary Table 3).

### Bleomycin-induced PF mouse model

Acute PF was induced in 8- to 10-week-old female C57BL/6 mice by intratracheal challenge with sterile PBS containing 2.5 U/kg bleomycin. Control animals received equal amounts of sterile PBS. Prophylactic treatment was started immediately and included three i.p. injections of 50 mg/kg m-tACPA, cIgG, or PBS at days 0, 2, and 5 after bleomycin challenge (Fig. 2a). Throughout the study, the animals were observed daily for clinical signs indicating a human endpoint and were weighed once every 2 days. Mice were terminated at day 14 or 21 after bleomycin challenge. Bronchoalveolar lavage fluid (BALF) was isolated after the mice were sacrificed on day 14 with instillation of 1 ml saline using a tracheal cannula. BALF was centrifuged at 500 × *g* for 100 min, and supernatants were stored at −80 °C until assayed for protein concentration using a BCA™ protein assay kit (Pierce). The cell pellet was removed and subjected to total and differential cell counts as previously described.^89^ On days 14 and 21, the sacrificed mice were subjected to midline thoracotomy. The trachea was removed, and the lungs were fixed by instillation of 2% buffered formalin for 24 h, followed by perfusion with 70% ethanol for another 24 h, before routine processing and paraffin embedding. Multiple sections from each lung were stained with H&E.

### DSS-induced colitis mouse model of IBD

Acute colitis was induced in 20–23 g male C57BL/6 mice by administering drinking water supplemented with 5% (w/v) DSS for 7 days. Mice in the control group received only regular drinking water. To initiate therapeutic treatment, 40 mg/kg m-tACPA or cIgG was injected i.p. on days 3 and 5 following the start of DSS consumption (Fig. 3a). Clinical scores were determined according to stool appearance score (0–3), stool blood score (0–2), and mouse appearance (0–2). On day 7 after DSS administration, the mice were sacrificed, and circulating neutrophils were assessed automatically using a Sysmex XT-2000iV hematology analyzer. The proximal colons were removed, fixed in 10% formalin and embedded in paraffin for subsequent periodic acid-Schiff staining to evaluate tissue damage (Supplementary Table 4).

### LPS-induced sepsis mouse model

To induce acute sepsis, LPS was injected i.p. at a dose of 10 mg/kg in 8-week-old male BALB/c mice. Within 30 min of LPS challenge, the mice were treated with a single i.v. injection of 50 mg/kg hz-tACPA or cIgG (Fig. 4a). Dexamethasone (Dex) was used as a reference compound and given orally at a dose of 10 mg/kg 1 h prior to LPS challenge. Survival was monitored over time, and the mice were terminated at 96 h after LPS injection. Lung, liver, kidney, and spleen tissues were collected, preserved in 10% buffered formalin and subsequently embedded in paraffin. Sections were stained with H&E or periodic acid-Schiff diastase for the assessment of tissue damage (Supplementary Table 5). Mice that died during the night and between monitoring were not sampled.

### Patient information and human neutrophil isolation

Buffy coats from HVs were obtained from the Sanquin blood bank in Nijmegen, The Netherlands. RA whole blood and SF samples were obtained from the Rheumatology Department at the Radboud University Medical Center in Nijmegen, The Netherlands, and SLE whole blood was obtained from the University College London Hospitals, UK. Blood samples were obtained from patients with a diagnosis of RA or SLE according to the criteria established by the American College of Rheumatology.

Neutrophils from HVs and RA and SLE patients were isolated by dextran-Ficoll^90^ or Histopaque-1077 and −1119 (Sigma-Aldrich) double-gradient density centrifugation.^91^ All preparations contained >95% neutrophils and had a viability of >95% as confirmed by flow cytometry using anti-human CD66b antibody (BioLegend) and Guava ViaCount reagent (EMD Millipore), respectively.

### Human NET induction and inhibition studies

Neutrophils from HVs and RA and SLE patients were seeded in 24-well plates at a density of 0.9 × 10^6^ cells/well in RPMI 1640 medium containing GlutaMAX and 1 mM CaCl_2_ supplemented with serum (1% fetal calf serum (FCS; Bodinco) or 2% serum from HVs) or 1% BSA, which was designated NET assay buffer. Prior to neutrophil stimulation, NET assay buffer alone or in combination with 25 µg/ml m-tACPA, hz-tACPA, anti-acH2A, anti-metH2A, anti-phosH2A, or cIgG was added. After a 15 min incubation at 37 °C and 5% CO_2_ in which neutrophils were allowed to settle, the cells were stimulated with 25 µM A23187 (Thermo Fisher Scientific), 2,5% RA SF, 4% gout SF, or thrombin-activated platelets (neutrophil/platelet ratio = 1:50) for 4 h at 37 °C and 5% CO_2_. Platelets derived from citrate blood of HVs were stimulated with HEPES buffer, pH 7.4 (134 mM NaCl, 2.9 mM KCl, 12 mM NaHCO_3_, 0.34 mM Na_2_PHO_4_, 1 mM MgCl_2_, 5 mM glucose, 5 mM HEPES, and 0.35% BSA), containing 0.01 U/ml recombinant thrombin (Calbiochem) for 15 min at 37 °C and 5% CO_2_.

### Human NET quantification through the measurement of DNA or MPO activity

After neutrophil stimulation, supernatants were aspirated, and cells, including NETs, were gently washed twice with NET assay buffer to remove non-NET-bound proteins, such as MPO. To harvest the NETs, the wells were incubated with NET assay buffer containing 15 U/ml S7 nuclease (Thermo Fisher Scientific). After a 15 min incubation at 37 °C and 5% CO_2_, 2 mM EDTA was added to stop the nuclease activity. The digests were transferred to a 96-well plate and centrifuged for 5 min at 20 × *g*. NET-containing supernatants were collected, and MPO activity was measured by incubating similar volumes of NET-containing supernatant with TMB substrate (Life Technologies) for 10 min at room temperature. H_2_SO_4_ was added to a final concentration of 300 mM to stop the reaction, and the optical density (OD) was measured at 450 nm.

To detect NETs by measuring DNA, NET-containing supernatant was transferred to a 96-well plate and incubated with a similar volume of NET assay buffer containing 5 µM Sytox Orange for 10 min at room temperature. Fluorescence was measured using an EnVision multilabel plate reader (Perkin Elmer) at 547/570 nm (excitation/emission). The MPO activity and amount of DNA are surrogate measures for the amount of NETs present.^92^

### NET detection by ELISA

To quantify MPO/DNA complexes, we performed a previously described protocol.^18^ In short, 5 µg/ml anti-human-MPO monoclonal antibody (Bio-Rad, 0400-0002) was coated in a flat-bottom 96-well ELISA plate overnight. After blocking with 1% BSA (w/v) in PBS, harvested NETs were added in combination with anti-DNA monoclonal antibody conjugated with peroxidase (Component 2 from the cell death detection ELISA kit; Roche, 11774425001) according to the manufacturer’s instructions. Samples were incubated for 2 h at room temperature while shaking at 320 rpm and were subsequently washed three times with PBS. Peroxidase substrate was added and incubated for 40 min at 37 °C in the dark, after which H_2_SO_4_ (300 mM final concentration) was added. Finally, NETs were detected by measuring the absorbance at 450 nm in a standard ELISA plate reader.

Citrullinated histone/MPO complexes were measured as follows. Flat-bottom 96-well ELISA plates were coated overnight at 4 °C with 5 µg/ml hz-tACPA. After blocking with 1% BSA (w/v) in PBS, harvested NETs were diluted four times, added to the ELISA plate and incubated for 2 h at room temperature while shaking at 200 rpm. After three washes with PBS, 0.2 µg/ml mouse anti-human MPO antibody was added and incubated for 1 h at room temperature. Subsequently, the wells were washed three times with PBS and incubated with 0.65 µg/ml HRP-labeled goat anti-mouse antibody (Dako, P0260) for 1 h at room temperature. After three washing steps with PBS, peroxidase substrate was added and incubated for 10 min at room temperature in the dark. Finally, H_2_SO_4_ (300 mM final concentration) was added, and the absorbance was measured at 450 nm in a standard ELISA plate reader.

### Live imaging immunofluorescence NET assay

The live imaging immunofluorescence NET assay and quantification were performed as described previously.^93^ In short, neutrophils from HVs were incubated in RPMI 1640 (without phenol red) supplemented with 2% FBS, 50 U/ml penicillin-streptomycin, 10 mM HEPES (referred to hereafter as RPMI-pr 2%), and Nuclear-ID Red DNA stain (diluted 1000×; Enzo Life Sciences, ENZ-52406) for 15 min at 37 °C. Neutrophils were seeded in 0.001% poly-L-lysine (Sigma-Aldrich, P4832) precoated clear bottom 96-well plates (Corning, Costar 3603) and challenged with 5 µM A23187 in the presence of 25 µg/ml full-length dc-tACPA or 17.7 µg/ml dc-tACPA F(ab′)2 antibody fragments (similar molarity as that of dc-tACPA full-length and F(ab′)2 antibody fragments). A23187 was resuspended in RPMI-pr 2% containing 160 nM Sytox Green (Life Technologies, S7020). NET release was measured at 37 °C and 5% CO_2_ in the IncuCyte ZOOM platform with a 20x objective for 240 min. Every 30 min, a set of three images (phase contrast, Sytox Green (Exc/Em: 504/523), and Nuclear-ID Red (566/650)) was taken.

For quantification of NETs, the images were processed with Fiji software (version 2.0.0-rc-69/1.52p). Thresholding for Nuclear-ID Red images was performed with the “Default (3900-1e30)” logarithm and Sytox Green images with the “Default (2200-1e30)” logarithm. NETs were determined as Sytox Green+ particles with a surface of >190 µm^2^.

### Immunofluorescence staining of human NETs

NET induction and inhibition studies with neutrophils from HVs were performed as described above, with 2 × 10^5^ neutrophils seeded onto H_2_SO_4_-etched coverslips. In short, after fixation with 4% paraformaldehyde (PFA, Merck), cells were stained with the appropriate antibodies, including 1 µg/ml mouse anti-NE monoclonal antibody (Santa Cruz Biotechnology, sc-55548) and 2 µg/ml rabbit anti-human citH3 polyclonal antibody (Abcam, ab5103). For secondary antibody incubations, 2 µg/ml goat anti-rabbit IgG Alexa Fluor 647 polyclonal antibody was first added (Invitrogen, A21245), washed thoroughly and then 1 µg/ml rabbit anti-mouse IgG Alexa Fluor 488 polyclonal antibody (Invitrogen, A11059) was used. DNA was counterstained with 10 µM DAPI (Sigma-Aldrich). Samples were mounted and visualized with spinning disk confocal microscopy (Revolution Confocal System, Ireland) with PLAPON 606 O/TIRFM-SP, NA 1.45 and UPLSAPO 100XO, NA 1.4 objectives (Olympus, Hamburg, Germany). The percentage of NET-releasing cells was determined by examining 200 neutrophils in a double-blinded experimental procedure.

On another occasion, NET-releasing neutrophils were stained with 4.4 µg/ml ch-tACPA in combination with a 4 µg/ml goat anti-human IgG (H + L) cross-adsorbed Alexa Fluor 488 antibody (Invitrogen, A-11013). To visualize m-tACPA, which was used to inhibit NET formation, 4 µg/ml goat anti-mouse IgG (H + L) cross-adsorbed Alexa Fluor 594 antibody (Invitrogen, A-11005) was used.

### Isolation of mouse neutrophils from bone marrow and immunofluorescence staining of mouse NETs

Neutrophils were isolated from the bone marrow of C57BL/6J mice by negative selection using the EasySep™ mouse neutrophil enrichment kit (Stemcell Technologies) according to the manufacturer’s instructions. The purity of isolated neutrophils was checked by flow cytometry using an anti-Ly6G antibody (Biolegend, 128046) and was >90%.

Isolated bone marrow neutrophils were adjusted to a concentration of 2 × 10^6^ cells/ml in HBSS containing calcium and magnesium. A total of 100 μl of neutrophil suspension (2 × 10^5^ cells) was added to each well of an 8-well chamber slide (Thermo Fisher Scientific). HBSS alone or together with 25 μg/ml dc-tACPA or cIgG was incubated with the neutrophils for 15 min before adding 150 μl of HBSS containing 1.12 µM A23187 or vehicle control. The chamber slide was incubated for 3 h at 37 °C and 5% CO_2_. Subsequently, 2% (v/v) PFA was added to each well, and the preparations were incubated for 12 h at 4 °C. The samples were blocked with 10% FCS (Biochrome) in PBS for 1 h at room temperature. Primary antibodies, including rabbit anti-citH3 (5 µg/ml; Abcam, ab5103) or TRITC-conjugated goat anti-human IgG (20 µg/ml; Jackson ImmunoResearch, 109-025-003), were added to PBS containing 10% FCS and incubated for 12 h at 4 °C. The slides were washed three times with PBS, and secondary Cy5-conjugated goat anti-rabbit IgG (3.75 µg/ml; Jackson ImmunoResearch, 111-175-144) was added and incubated for 1.5 h at room temperature in the dark. The slides were again washed with PBS. Staining solution containing 2.5 µM Hoechst or 2.5 µM Sytox Green in PBS was added and incubated for 15 min at room temperature. After washing with PBS, the samples were embedded in mounting medium (BIOZOL). The slides were analyzed on a BZ-X710 microscope (Keyence), and NETs (colocalization of Hoechst and citH3) were quantified by Fiji imaging software (version 2.0.0-rc-69/1.52i). In short, colocalization of Hoechst and citH3 staining was detected using the colocalization threshold plugin and converted to an 8-bit grayscale. The amount of NETs (large extracellular filaments) was calculated and is represented as the percentage NET area per image.

### Immunofluorescence staining of NETs from the pristane-induced peritonitis mouse model

To determine mouse NET-inhibitory capacity of tACPA in vivo, a previously described pristane-induced peritoneal cell influx mouse model was used.^36^ In brief, 50 mg/kg cIgG or dc-tACPA was injected i.p. immediately after injection of 500 µl pristane oil (Sigma-Aldrich), followed by a second injection of 50 mg/kg cIgG or dc-tACPA 12 h later. After a total of 24 h, inflammatory cells were isolated from the peritoneum, and the composition of the cell infiltrates was analyzed by FACS as described below. Inflammatory cells were adjusted to 1 × 10^6^ cells/ml and transferred to either flow chamber slides or cytospin slides. Cells were fixed with 2% (v/v) PFA and subsequently blocked with PBS + 10% fetal calf serum (FCS). Staining and analysis were performed as described above with rabbit anti-NE antibody (1:200; Abcam, ab21595) or TRITC-conjugated goat anti-human IgG (20 µg/ml; Jackson ImmunoResearch, 109-025-003). To visualize macrophages, we used AF488-conjugated rat anti-mouse F4/80 antibody (2.5 µg/ml; Biolegend, 123120). After washing with PBS, incubation with Cy5-conjugated goat anti-rabbit IgG (3.75 µg/ml; Jackson ImmunoResearch, 111-175-144) was performed.

### FACS analysis of peritoneal lavage fluid from the pristane-induced peritonitis mouse model

Isolated peritoneal cells were washed with PBS and stained with the following fluorescent probes obtained from BioLegend: BV570-conjugated anti-CD11b (0.66 µg/ml; 101233), APC/Fire750-conjugated anti-Ly6c (0.67 µg/ml; 128046), PE/Cy7-conjugated anti-Ly6g (0.5 µg/ml; 127617), AF488-conjugated anti-F4/80 (1 µg/ml; 123120), APC-conjugated anti-B220 (0.5 µg/ml; 103212), PerCP/Cy5.5-conjugated anti-CD3 (0.67 µg/ml; 100217), and AF700-conjugated anti-CD11c (1.67 µg/ml; 117319). Flow cytometry analysis of peritoneal cells was performed on a Gallios flow cytometer (Beckman Coulter). The data were analyzed using Kaluza 2.1 software (Beckman Coulter), and electronic compensation was applied to eliminate bleed-through fluorescence. Cell subsets were then determined via receptor expression and FSc/SSc exclusion as follows: B cells (B220^+^), T cells (CD3^+^), granulocytes (CD11b^+^, Ly6c^+^, Ly6g^+^), small macrophages (CD11b^+^, Ly6c^+^, Ly6g^−^, F4/80^int^, smaller FSc/SSc), and large macrophages (CD11b^+^, Ly6c^+^, Ly6g^−^, F4/80^high^, larger FSc/SSc). Cells that were negative for all markers were described as “unidentified cells”.

### CIA mouse model of IA

To induce chronic IA, bovine collagen II was diluted to a concentration of 2 mg/ml in 50 mM acetic acid and emulsified in equal volumes of Freund’s complete adjuvant. On day 0, 10- to 12-week-old male DBA/J1 mice were immunized intradermally at the tail base with 100 µg bovine CII. On day 21, the mice received i.p. booster injections of 50 µg bovine CII dissolved in PBS, and the onset of arthritis occurred a few days later (Fig. 7a). The mice were considered to have arthritis when significant changes in redness and/or swelling were noted in the digits or in other parts of the paws. Joint inflammation in each paw was scored as described above (CAIA mouse model of IA). Therapeutic treatment was started early after the onset of disease (between days 21–28) when the mean arthritis score (MAS) was ≥0.75 on an arbitrary scale of 0–8 (0–2 per paw) and included four repeated intravenous (i.v.) injections 4 days apart with the indicated doses of hz-tACPA or cIgG. The mice were terminated at day 14 after the start of treatment. The ankle and knee joints were collected and stored in formalin for histological analysis.

### Immunofluorescence staining of NETs in paw sections of CIA mice

For a histological overview, mouse hind paw sections were stained for 5 min with 1:2 diluted EMD Millipore^TM^ Mayer’s Hemalum solution (EMD Millipore, 1092492500) and 0.5% eosin Y (Roth, X883.2) in Aqua dest. For immunofluorescence, the hind paw sections were blocked for 1 h with 5% normal horse serum blocking solution (Vector Laboratories, S-2000) in PBS. Subsequently, the sections were stained overnight at 4 °C in 5% normal horse serum blocking solution in PBS with the following primary antibodies: rat anti-mouse Ly6G (10 µg/ml; BD Biosciences, 551459), polyclonal rabbit anti-citH3 (4 µg/ml; Abcam, ab5103), and goat anti-mouse MPO (5 µg/ml; R&D Systems, AF3667). Following extensive washing with PBS, the sections were stained for 1 h at RT in 5% normal horse serum blocking solution in PBS with the secondary antibodies, which were obtained from Thermo Fisher Scientific: DyLight 488-conjugated donkey anti-goat IgG (H + L) (1 µg/ml; SA5-10086), DyLight 550-conjugated donkey anti-rat IgG (H + L) (1 µg/ml; SA5-10027), and DyLight 650-conjugated donkey anti-rabbit IgG (H + L) (1 µg/ml; SA5-10041). To visualize the nucleus, sections were counterstained with 10 µM DAPI and thereafter mounted with ProLong Gold antifade mounting reagent (Thermo Fisher Scientific, P10144). Images were acquired on a DMi8 microscope with a motorized stage (Leica) using a 20x objective and LASX Navigator software (Leica), and neutrophils and NETs were quantified by Fiji imaging software.

### Radiological analysis

X-ray images of ankles and knees of the hind paws were taken using a Faxitron MX20 instrument and analyzed using Faxitron DX 1.0 software with ImageAssist. Radiographic destruction was scored on an arbitrary scale of 0–5 (0 = joints without pathology; 5 = joints with maximal pathology). Scoring was performed in a double-blinded manner.

### Histological analysis

Histological analysis was performed as described previously.^94^ In short, joints from the hind paws were fixed for at least 4 days in 4% PFA, decalcified in 5% formic acid, and subsequently dehydrated and embedded in paraffin. Standard frontal sections of 7 µm were mounted on SuperFrost slides. H&E staining was performed to study joint inflammation and synovial-like lining formation. The severity of inflammation was scored on a scale of 0–3 (0 = no cells, 1 = mild cellularity, 2 = moderate cellularity, and 3 = maximal cellularity). To study proteoglycan (PG) depletion from the cartilage matrix, sections were stained with SO followed by counterstaining with fast green (BDH Chemicals). Depletion of PG was determined using an arbitrary scale of 0–3 (0 = normal, fully intact cartilage; 3 = PG-depleted cartilage). Chondrocyte death was scored on a scale of 0–3 (0 = no loss of chondrocyte nuclei; 3 = completely empty cartilage surface). Cartilage and bone erosion were graded on a scale of 0–3 (0 = no damage; 3 = complete loss of cartilage or bone structure). Histopathological changes in the joint were scored on three semi-serial sections of the joint that were spaced 70 µm apart. Scoring was performed in a double-blinded manner.

Human RA synovial tissue was collected during joint replacement surgery in culture medium with antibiotics and freshly processed into standardized biopsies with a diameter of 6 mm using disposable skin biopsy punches (Staffel). The tissue was directly embedded in OCT compound (Tissue-Tek) and snap-frozen in liquid nitrogen, and cryosections of 6 μm were prepared on SuperFrost slides (Menzel-Gläser) for immunohistochemistry. Cryosections were first dried in air for 30 min. Next, the cryosections were fixed in 4% PFA for 15 min and incubated with 1% H_2_O_2_ in methanol for another 15 min. Subsequently, the sections were stained with 1.7 µg/ml m-tACPA or cIgG for 1 h in PBS containing 5% normal rabbit serum. Subsequently, the sections were washed with PBS and incubated with 1.3 µg/ml HRP-conjugated rabbit anti-mouse antibody (DAKO, P0260) for 1 h in PBS containing 5% normal rabbit serum. After washing in PBS, peroxidase was developed with diaminobenzidine substrate for 10 min. Finally, the sections were counterstained with hematoxylin for 1 min, dehydrated, and closed in Permount.

### Study approval

All procedures regarding CAIA and CIA mouse experiments were approved by the Institutional Animal Care and Use Committee and conformed to the guidelines of the Dutch Council of Animal Care. All procedures regarding the bleomycin-induced PF mouse model were approved by the Institutional Animal Care and Use Committee and conformed to the guidelines of the NIH. The CRO Fidelta in Zagreb, Croatia, performed DSS-induced colitis and LPS-induced sepsis mouse models under their specific animal welfare guidelines and law NN 081-99-266/1 of 9 February 1999. The pristane-induced peritonitis mouse experiments were approved by the ethical committee of the University of Erlangen-Nürnberg, Germany. All HVs and RA and SLE patients gave informed consent in accordance with the Declaration of Helsinki.

### Statistics

All statistical analyses were performed using GraphPad Prism software version 6. The results are reported as the mean ± standard error of the mean (SEM) and were considered significant at *P* < 0.05. The normal distribution of each data set was assessed with the D’Agostino-Pearson omnibus normality test. The comparison of two different groups was performed by two-tailed Mann–Whitney test or unpaired two-tailed Student’s *t* test. To compare three or more groups, one- or two-way analysis of variance (ANOVA) with Tukey’s multiple comparisons test, one-way ANOVA with post hoc Dunn’s test for Kruskal–Wallis multiple comparisons test, or two-way ANOVA with Dunnett’s multiple comparisons test were performed. Spearman’s nonparametric test or Pearson’s correlation test was used to calculate the correlations between two groups. The number of sampled units, *n*, is indicated in the figure legends.

## Supplementary information

Chirivi et al Supplemental data
