# Supplementary material for: Therapeutic ACPA inhibits NET formation: a potential therapy for neutrophil-mediated inflammatory diseases
Source: Cell Mol Immunol. 2020 Mar 20;18(6):1528–44. doi: 10.1038/s41423-020-0381-3 (PMC8166830; doi:10.1038/s41423-020-0381-3)
Supplement: Supplementary file 1 — Chirivi et al Supplemental data [file 41423_2020_381_MOESM1_ESM.pdf]

Supplementary Figures

a

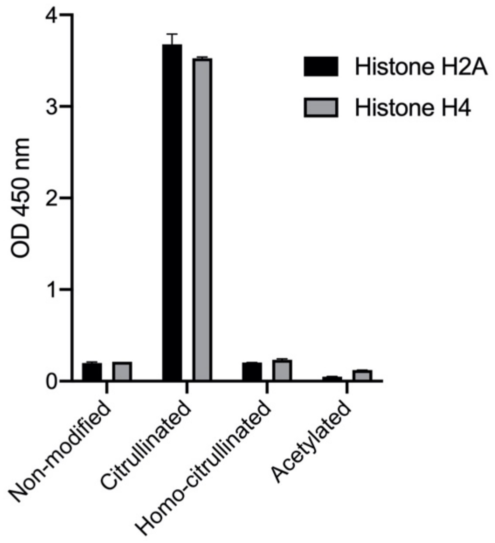

b

**Non-modified**	
H2A	S G R G K Q G G K A R A K (biotin)
H4	S G R G K G G K G L G K K (biotin)
**Citrullinated**	
H2A	S G R(Cit) G K Q G G K A R A K (biotin)
H4	S G R(Cit) G K G G K G L G K K (biotin)
**Homo-citrullinated**	
H2A	S G R G K(homoCit) Q G G K A R A K (biotin)
H4	S G R G K(homoCit) G G K G L G K K (Biotin)
**Acetylated**	
H2A	S G R G K(Ac) Q G G K A R A K A K S R S S R A G G K (Biotin)
H4	S G R G K(Ac) G G K(Ac) G L G K(Ac) G G A K(Ac) R H R K V G G K (Biotin)

Supplementary Figure 1 tACPA specifically binds to citrullinated N-terminal histone H2A and H4 peptides. (**a**) Non-modified, citrullinated, homo-citrullinated, and acetylated N-terminal histone H2A and H4 peptides (100 ng/well) were used to test hz-tACPA (10 µg/ml) binding (n = 2). Results depicted as means ± SEM. (**b**) Peptide sequences used in the ELISA.

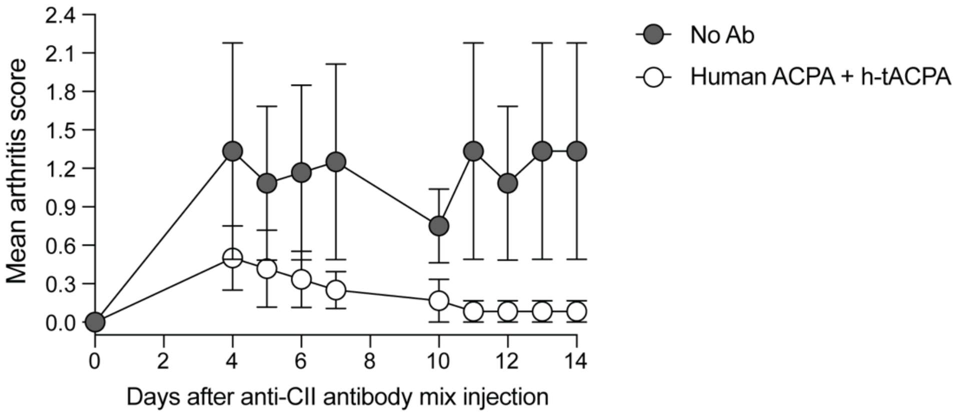

Supplementary Figure 2 tACPA is able to perform its therapeutic activity in the presence of human ACPA. Mice were injected with a low dose (1.6 mg) of anti-CII antibody mix followed by LPS at day 3 to induce acute inflammatory arthritis. Antibody (Ab) treatment started directly after LPS injection by administration of a mix of h-tACPA (50 mg/kg) and a human ACPA (50 mg/kg), or only PBS (No Ab). Mice were terminated at day 14. The mean arthritis score (MAS) of CAIA mice was evaluated for 14 days (n = 3 mice per group). Results depicted as means ± SEM.

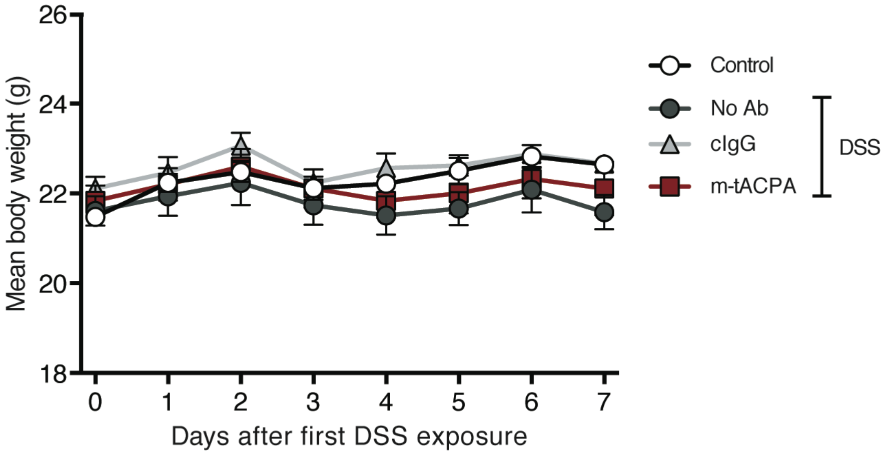

Supplementary Figure 3 Mean body weight of DSS-induced colitis mice. Mice were challenged with normal drinking water (Control) or drinking water containing 5% (w/v) DSS for 7 days and injected with PBS (No Ab), 50 mg/kg cIgG, or m-tACPA at day 3 and 5. Mice were terminated at day 7. The mean body weight of DSS-induced colitis mice was evaluated for 7 days (*n* = 5-8 mice per group). Results depicted as means ± SEM.

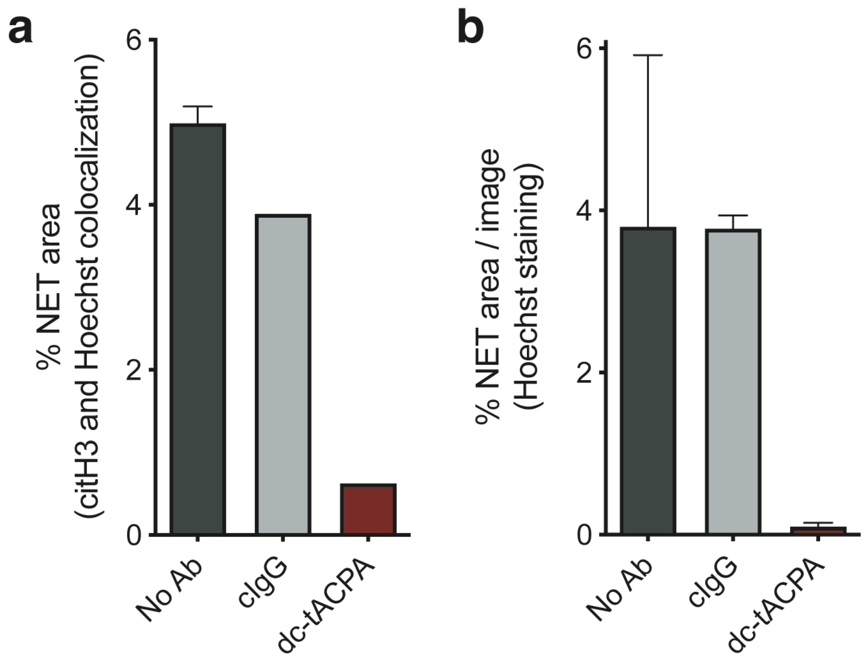

Supplementary Figure 4 tACPA inhibits A23187-induced NET formation in mouse BM-derived neutrophils *in vitro.* BM-derived mouse neutrophils were stimulated with A23187 in the absence (No Ab) or presence of 25 µg/ml dc-tACPA or cIgG. Quantification of NETs was performed with either (**a**) colocalization of citH3 and Hoechst (*n* = 1-2) or (**b**) Hoechst alone (*n* = 2). Results depicted as means ± SEM.

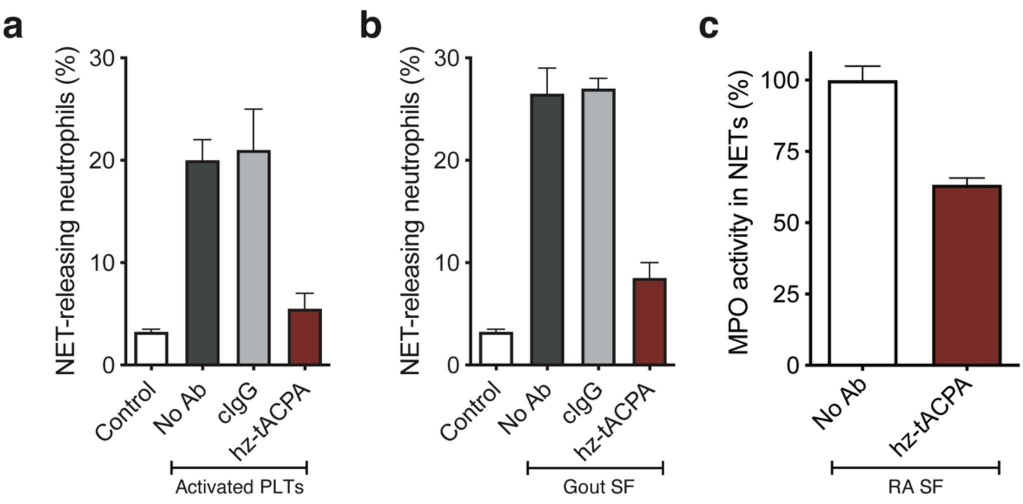

Supplementary Figure 5 Inhibition of NET release in response to activated PLTs, gout SF, or RA SF. Quantification of microscopic images from NET release induced by (**a**) human activated platelets (PLTs) and (**b**) gout synovial fluid (SF) without antibody treatment (No Ab) or in the presence of 25 µg/ml cIgG or hz-tACPA (n = 2). Non-stimulated neutrophils (Control) were used to set the background of NET release. The percentage of NET-releasing cells was determined by examining 200 neutrophils. (**c**) Neutrophils from healthy volunteers (HV) were stimulated with 2,5% RA SF from a CCP+ patient in the absence (No Ab) or presence of 25 µg/ml hz-tACPA. MPO activity in NETs was measured (n = 1). The mean of No Ab was set at 100% to calculate the percentages. Results depicted as means ± SEM.

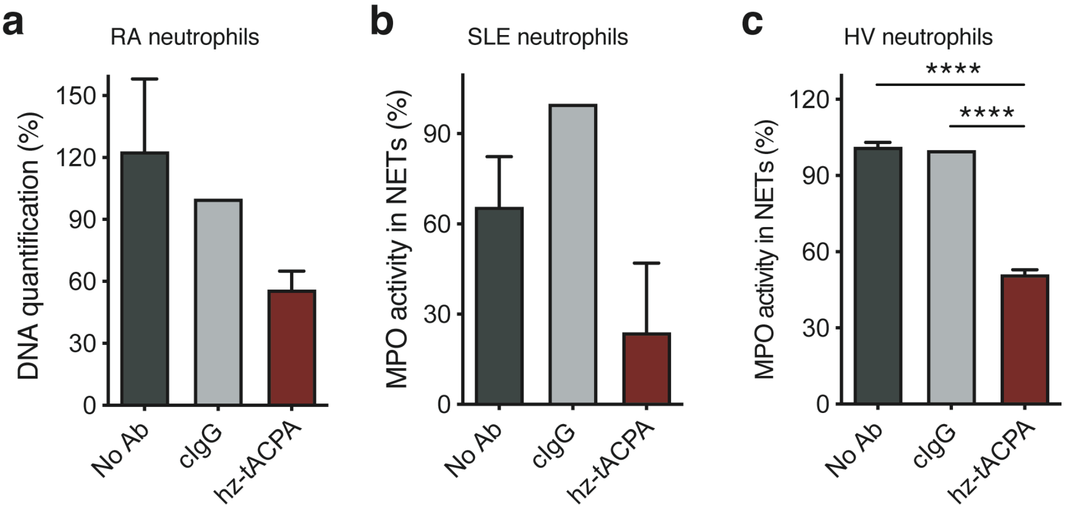

Supplementary Figure 6 Inhibition of NET release in neutrophils from healthy volunteers (HVs), and RA and SLE patients. Neutrophils from HVs, and RA and SLE patients were stimulated with 25 µM A23187 in the absence (No Ab) or presence of 25 µg/ml cIgG or tACPA. NET release in (**a**) RA and (**b**) SLE neutrophils was inhibited with hz-tACPA, while NET release in (**c**) HV neutrophils was inhibited with different tACPA molecules, including m-tACPA, ch-tACPA, hz-tACPA, and dc-tACPA. The presence of DNA (**a**) or the activity

of MPO in NETs (**b** and **c**) was measured (*n* = 2 for RA, *n* = 3 for SLE, and *n* = 109 for HV). Results depicted as means ± SEM. The mean of cIgG was set at 100% in each individual experiment to calculate the percentages. ****P<0.0001 using one-way ANOVA with Tukey's multiple comparisons test.

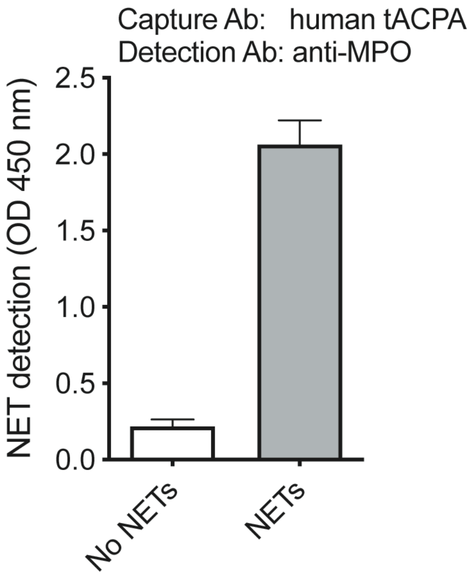

Supplementary Figure 7 tACPA binds to NETs in a sandwich ELISA. Supernatant of non-stimulated neutrophils (No NETs) or A23187-triggered neutrophils (NETs) from healthy volunteers were isolated and used for a sandwich ELISA to confirm the binding of tACPA to NETs. hz-tACPA (25 µg/ml) was coated onto an ELISA plate to capture NETs from the supernatant. Subsequently, NETs were detected with a mouse anti-MPO antibody (1 µg/ml) followed by a secondary rabbit anti-mouse-HRP antibody (0.65 µg/ml) (*n* = 2). Results depicted as means ± SEM.

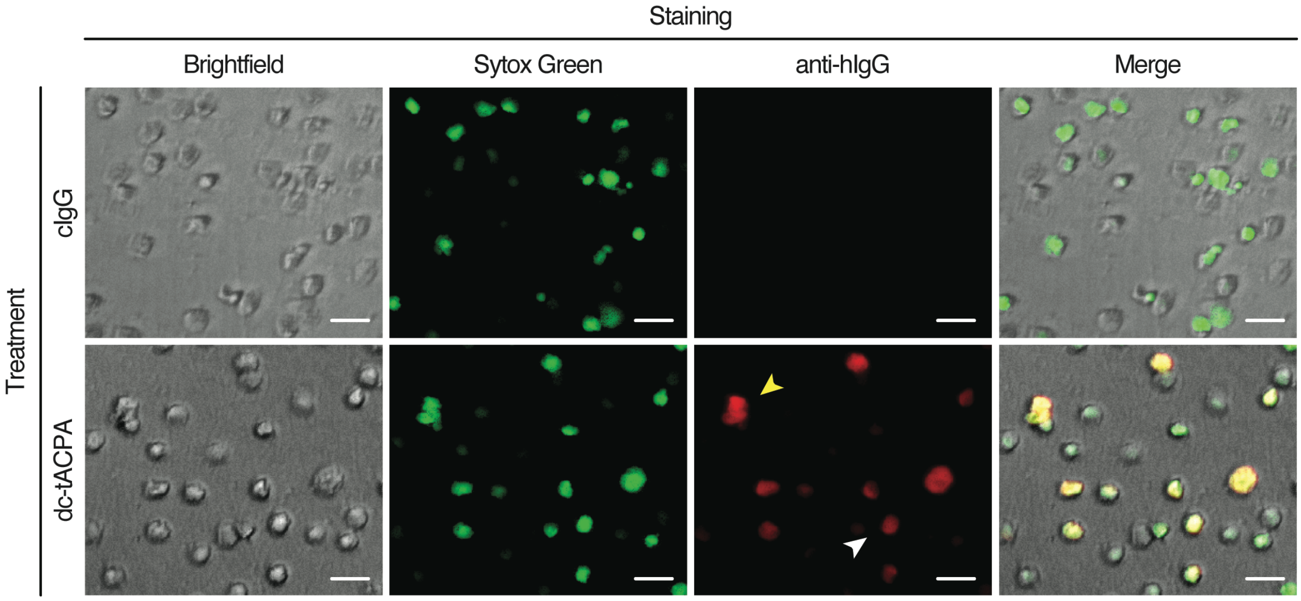

Supplementary Figure 8 tACPA binds to mouse (pre-)NETs *in vitro.* BM-derived mouse neutrophils were stimulated with A23187 in the absence (No Ab) or presence of 25 µg/ml dc-tACPA or cIgG. Representative images of dc-tACPA binding to pre-NETs (white arrowhead) as well as expelled NETs (yellow arrowhead) visualized with transmitted white light (Brightfield), Sytox Green, and an anti-hIgG-TRITC antibody. Scale bars: 20 µm.

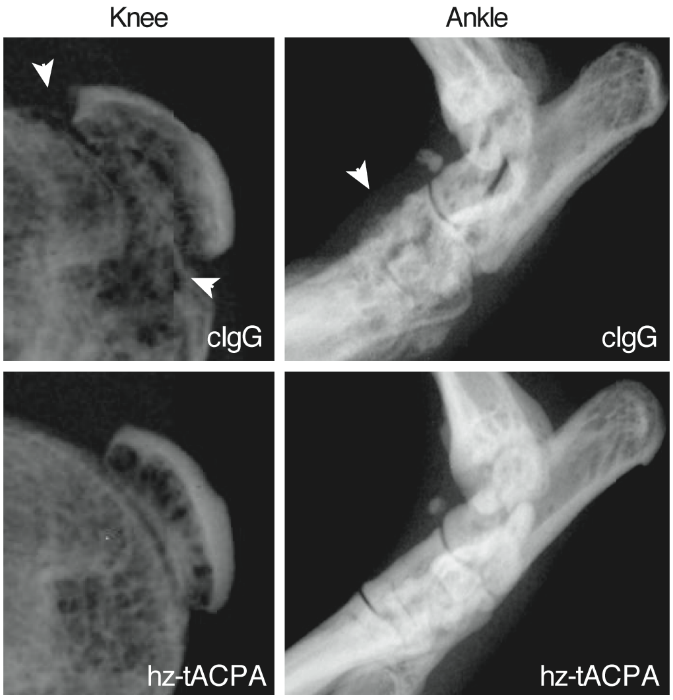

Supplementary Figure 9 X-ray of knees and ankles from CIA mice. To induce chronic IA, mice were injected day 0 and 21 with CII. Therapeutic treatment includes four injections with tapered dosing regimens of 50/50/50/50 mg/kg cIgG or 50/50/50/15 mg/kg hz-tACPA, and started after onset of the disease (between day 21-28) when the MAS was ≥0.75. Representative X-ray images from knees and ankles of cIgG- and hz-tACPA-treated mice at day 14 after the first antibody injection. Bone erosion (white arrowheads) was only observed in the joints of cIgG-treated mice.

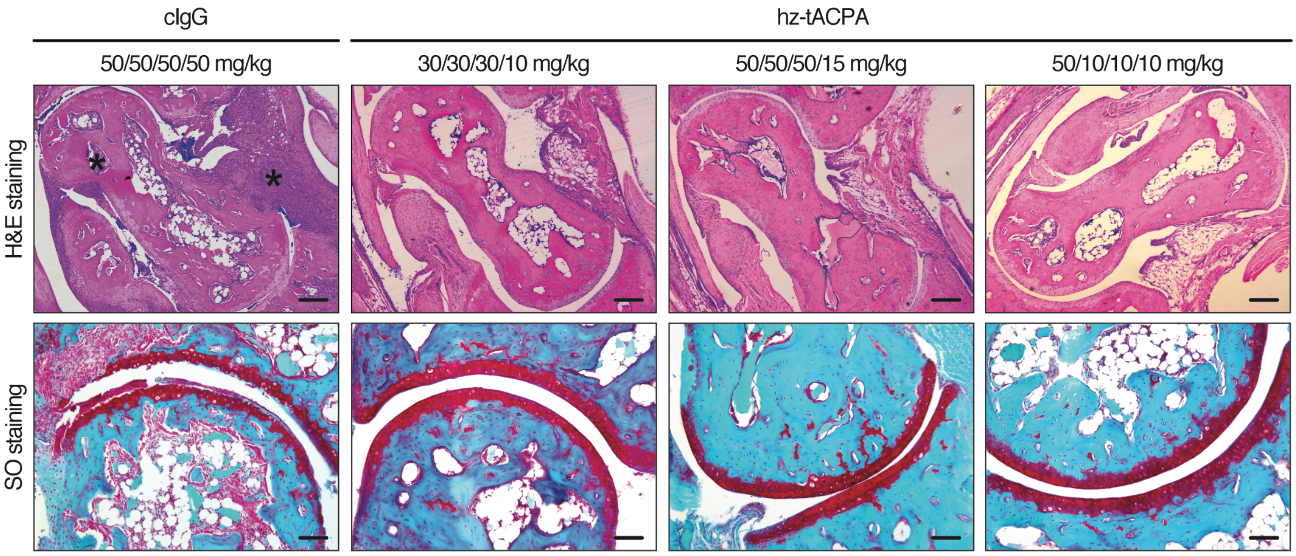

Supplementary Figure 10 H&E and SO staining of ankle joints from CIA mice. To induce chronic IA, mice were injected on day 0 and on day 21 with CII. Therapeutic treatment includes four injections with tapered dosing regimens of 50/50/50/50 mg/kg cIgG or hz-tACPA (30/30/30/10, 50/50/50/15, or 50/10/10/10 mg/kg), and started after onset of the disease (between day 21-28) when the MAS was ≥0.75. Images represent H&E (upper panel) and SO (lower panel) staining of ankle joints from cIgG- and hz-tACPA-treated mice at day 14 after the first antibody injection. Inflammatory cell influx (asterisks) was only observed in the joints of cIgG-treated mice. Scale bars: 100 μm.

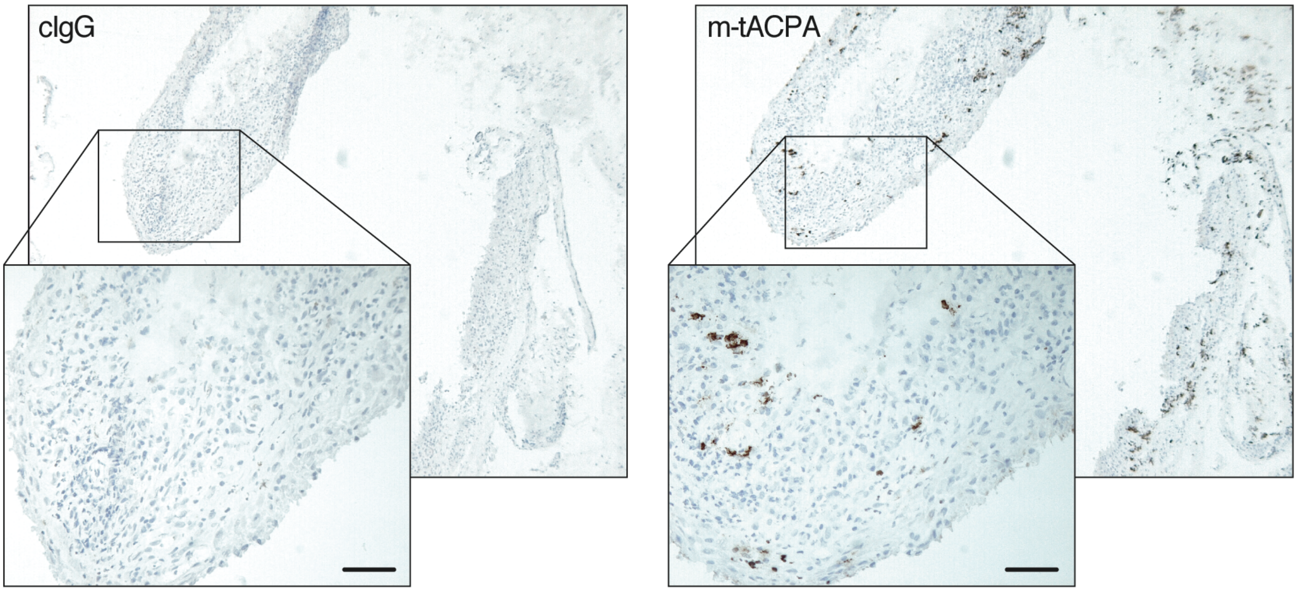

Supplementary Figure 11 Binding of tACPA to human RA synovial tissue. Representative images from human RA synovial tissue stained with 2 µg/ml cIgG or 0.125 µg/ml m-tACPA followed by an H&E staining. Tissue was collected during joint replacement surgery. Neutrophils and neutrophilic remnants were positively stained for tACPA. Scale bars: 100 µm.

Supplementary Tables

Macroscopical signs of inflammation	Score
1-2 Swollen toes	0.25
3-4 Swollen toes	0.50
Slightly swollen footpad or ankle	0.50-0.75
Swollen footpad or ankle	1.00
Swollen toes + slightly swollen footpad	1.25
Swollen toes + swollen footpad	1.5
Swollen footpad + swollen ankle	2.00

Supplementary Table 1 Scoring system for macroscopical signs of inflammation in CAIA and CIA mice. CAIA and CIA mice were considered to have arthritis when significant changes of redness and/or swelling were noted in the digits or in other parts of the paws. Joint inflammation in each paw was scored visually using a scale of 0-2 per paw (0 = not inflamed, 1 = mild inflammation, 1.5 = marked inflammation, and 2 = severe inflammation) with a maximal score of 8 per animal.

#	Name	Histone	Modification	Peptide sequence
1	H2A + H4	H2A<br>H4	No modification<br>No modification	S G R G K Q G G K A R A<br>S G R G K G G K G L G K G G A K R H R K V L R
2	citH2A	H2A	Citrullination (R3)	S G R(cit) G K Q G G K A R A
3	citH4	H4	Citrullination (R3)	S G R(cit) G K G G K G L G K G G A K R H R K V L R
4	phosH2A +<br>phosH4	H2A<br>H4	Phosphorylation (S1)<br>Phosphorylation (S1)	S(phos) G R G K Q G G K A R A<br>S(phos) G R G K G G K G L G K G G A K R H R K V L R
5	acH2A +<br>acH4	H2A<br>H4	Acetylation (K5, K9)<br>Acetylation (K5, K8, K12, K16)	S G R G K(ac) Q G G K(ac) A R A<br>S G R G K(ac) G G K(ac) G L G K(ac) G G A K(ac) R H R K V L R
6	sdmH4 +<br>admH4	H4<br>H4	Symmetric methylation (R3)<br>Asymmetric methylation (R3)	S G R(sdm) G K G G K G L G K G G A K R H R K V L R<br>S G R(adm) G K G G K G L G K G G A K R H R K V L R

Supplementary Table 2 H2A and H4 peptides used for immunization of CAIA mice. To determine the potential of citrullinated H2A and H4 as therapeutic target in a CAIA mouse model of RA, different N-terminal post-translationally-modified H2A and H4 peptides were used to immunize DBA/J1 mice prior to arthritis induction with an anti-CII antibody mix.

H2A + H4-immunized mice

Mice	Binding to H2A	Binding to H4	Selected for CAIA experiment
1	0.139	0.112	
2	0.876	0.641	X
3	0.090	0.070	
4	1.036	0.433	X
5	0.894	0.346	X

citH4-immunized mice

Mice	Binding to citH2A	Binding to H4	Selected for CAIA experiment
1	0.228	0.270	
2	0.151	0.372	
3	0.228	0.082	X
4	0.156	0.074	X
5	0.431	0.118	X

citH2A-immunized mice

Mice	Binding to citH2A	Binding to H2A	Selected for CAIA experiment
1	0.193	0.131	
2	0.143	0.084	
3	0.130	0.073	
4	1.363	0.29	X
5	0.544	0.134	
6	0.151	0.172	
7	0.099	0.086	
8	1.492	0.639	X
9	0.449	0.675	
10	2.291	0.729	X
11	2.362	0.491	X

phosH2A + phosH4-immunized mice

Mice	Binding to phosH2A	Binding to H2A	Selected for CAIA experiment
1	0.120	0.094	
2	0.302	0.088	X
3	0.128	0.112	
4	0.289	0.099	X
5	0.457	0.086	X
6	0.097	0.093	
7	0.048	0.093	
8	0.700	0.104	X
9	0.414	0.098	X

acH2A + acH4-immunized mice

Mice	Binding to acH2A	Binding to H2A	Selected for CAIA experiment
1	1.211	0.992	
2	1.213	0.330	X
3	1.205	0.151	X
4	1.043	0.152	X
5	1.349	0.182	X
6	1.376	0.117	X
7	1.332	0.112	X
8	0.135	0.128	

sdmH4 + admH4-immunized mice

Mice	Binding to admH4	Binding to sdmH4	Binding to H4	Selected for CAIA experiment
1	0.394	0.429	0.472	
2	0.771	0.812	0.073	X
3	0.105	0.129	0.101	
4	1.282	1.393	0.133	X
5	0.176	0.242	0.059	
6	0.953	1.365	0.315	X
7	0.145	0.166	0.176	
8	0.531	0.573	0.230	X
9	0.654	0.749	0.862	

Supplementary Table 3 Peptide-specific immune response in immunized DBA/J1 mice prior to arthritis induction. To determine the potency of citrullinated H2A (citH2A) and H4 (citH4) as therapeutic target in a CAIA mouse model of IA, different N-terminal post-translationally-modified H2A and H4 peptides were used to immunize DBA/J1 mice prior to arthritis induction with an anti-CII antibody mix. Peptide-specific immune response was determined in serum (1:100 diluted in PBS) using an ELISA. Since the first 5 amino acids of the N-terminus of H2A and H4 are identical, they can both be used to monitor an anti-citrulline response to both citH2A and citH4. Mice that developed a specific immune response against the modified peptides, but not the non-modified peptides, were selected for inclusion in the CAIA experiment. Results depicted as OD (450 nm) measurements.

Score	Severity of inflammation	Layers involved	Epithelial damage	Extent
0	None	No inflammation	Intact epithelium	No lesions
1	Mild	Inflammation in mucosa	Disruption of architecture	Punctuate
2	Moderate	Inflammation in mucosa and submucosa	Ulceration	Multifocal
3	Severe	Transmural inflammation	Ulceration	Diffuse

Supplementary Table 4 Scoring system for tissue damage in the proximal colon of DSS-induced colitis mice. After 7 days of DSS-induced colitis, the proximal colons were removed. Histological Periodic Acid Schiff-stained tissue sections of the proximal colon were scored for inflammation in the mucosa and submucosa, and transmural inflammation with a maximal score of 3 per animal.

Organ	Parameters	Maximum score
Lung	Oedema (0-3)<br>Congestion (0-3)<br>Hemorrhage (0-3)<br>Thickening of the septa (0-3)<br>Interstitial leukocyte infiltration (0-3)	15
Liver	Apoptotic bodies (0-1)<br>Enlarged sinusoids (0-3)<br>Hepatocyte necrosis (0-3)<br>Kupffer cell hyperplasia (0-3)<br>Kupffer cell hypertrophy (0-3)<br>Luminal leukocyte infiltration (0-3)<br>Loss of glycogen within hepatocyte (0-3)<br>Increased volume of endothelial cells (0-3)	22
Kidney	Erythrocyturia (0-1)<br>Tubular damage (0-3)	4
Spleen	Congestion (0-3)<br>Tingible bodies (0-1)<br>Accumulation of neutrophils (0-3)	7

Supplementary Table 5 Scoring system for tissue damage in the lung, liver, kidney, and spleen in LPS-induced sepsis mice. After 4 days of LPS-induced sepsis, lung, liver, kidney, and spleen were removed. Histological H&E-stained lung and liver tissue sections, Periodic Acid Schiff (PAS)-stained kidney sections, and PAS-diastase-stained liver sections were scored for specific parameters as shown in the table, with maximal scores of 15, 22, 4, and 7 for lung, liver, kidney, and spleen, respectively.

Materials and Methods

ELISA to test tACPA cross-reactivity

96-well plates (Greiner Bio-One, 655061) were coated with 100 ng/well neutravidin (Invitrogen, 31000) in PBS overnight at 4°C. Between every following step, the wells were washed with PBS containing 0.05% Tween 20 (PBS/Tween). After washing, the plates were blocked with 1% BSA (Sigma Aldrich, A3912) in PBS/Tween (BSA/PBS/Tween) for 60 min at room temperature, after which 100 ng/well non-modified, citrullinated, homo-citrullinated, or acetylated H2A or H4 peptides in BSA/PBS/Tween were coated for 60 min at 37°C. Subsequently, 10 µg/ml hz-tACPA in BSA/PBS/Tween was added to the wells and incubated for 60 min at room temperature. This step was followed by adding HRP-conjugated goat anti-human IgG (Southern Biotech, 2040-05; 4000x diluted) and after 60 min incubation at room temperature, wells were washed three times with PBS/Tween followed by three washes with PBS. The reaction was visualized with 100 µl TMB (Thermo Fisher, SB02) and subsequently stopped with 50 µl 2M H_2SO_4 after 5 min. The absorbance was measured at 450 nm using the Tecan Infinite F50.
